# Association of Malaria Infection During Pregnancy With Head Circumference of Newborns in the Brazilian Amazon

**DOI:** 10.1001/jamanetworkopen.2019.3300

**Published:** 2019-05-03

**Authors:** Jamille Gregório Dombrowski, Rodrigo Medeiros de Souza, Flávia Afonso Lima, Carla Letícia Bandeira, Oscar Murillo, Douglas de Sousa Costa, Erika Paula Machado Peixoto, Marielton dos Passos Cunha, Paolo Marinho de Andrade Zanotto, Estela Bevilacqua, Marcos Augusto Grigolin Grisotto, Antonio Carlos Pedroso de Lima, Julio da Motta Singer, Susana Campino, Taane Gregory Clark, Sabrina Epiphanio, Lígia Antunes Gonçalves, Cláudio Romero Farias Marinho

**Affiliations:** 1 Department of Parasitology, Institute of Biomedical Sciences, University of São Paulo, São Paulo, Brazil; 2 Multidisciplinary Center, Federal University of Acre, Acre, Brazil; 3 Department of Microbiology, Institute of Biomedical Sciences, University of São Paulo, São Paulo, Brazil; 4 Department of Cell and Developmental Biology, Institute of Biomedical Sciences, University of São Paulo, São Paulo, Brazil; 5 CEUMA University, Maranhão, Brazil; 6 Department of Statistics, Institute of Mathematics and Statistics, University of São Paulo, São Paulo, Brazil; 7 Faculty of Infectious and Tropical Diseases, London School of Hygiene & Tropical Medicine, London, United Kingdom; 8 Faculty of Epidemiology and Population Health, London School of Hygiene & Tropical Medicine, London, United Kingdom; 9 Department of Clinical and Toxicological Analyses, School of Pharmaceutical Sciences, University of São Paulo, São Paulo, Brazil

## Abstract

**Importance:**

Malaria during pregnancy is associated with adverse events for the fetus and newborn, but the association of malaria during pregnancy with the head circumference of the newborn is unclear.

**Objective:**

To investigate the association of malaria during pregnancy with fetal head growth.

**Design, Setting, and Participants:**

Two cohort studies were conducted at the general maternity hospital of Cruzeiro do Sul (Acre, Brazil) in the Amazonian region. One cohort study prospectively enrolled noninfected and malaria-infected pregnant women who were followed up until delivery, between January 2013 and April 2015. The other cohort study was assembled retrospectively using clinical and malaria data from all deliveries that occurred between January 2012 and December 2013. Data analyses were conducted from January to August 2017 and revised in November 2018. Clinical data from pregnant women and anthropometric measures of their newborns were evaluated. A total of 600 pregnant women were enrolled through volunteer sampling (prospective cohort study), and 4697 pregnant women were selected by population-based sampling (retrospective cohort study). After application of exclusion criteria, data from 251 (prospective cohort study) and 232 (retrospective cohort study) malaria-infected and 158 (prospective cohort study) and 3650 (retrospective cohort study) noninfected women were evaluated.

**Exposure:**

Malaria during pregnancy.

**Main Outcomes and Measures:**

The primary end point was the incidence of altered head circumference in newborns delivered from malaria-infected mothers compared with that from noninfected mothers. Secondary end points included measures of placental pathology relative to newborn head circumference.

**Results:**

In total, 4291 maternal-child pairs were analyzed. Among 409 newborns in the prospective cohort study, the mothers of 251 newborns had malaria during pregnancy, infected with *Plasmodium vivax*, *Plasmodium falciparum*, or both. Among 3882 newborns in the retrospective cohort study, 232 were born from mothers that had malaria during pregnancy. The prevalence of newborns with a small head (19 [30.7%] in the prospective cohort study and 30 [36.6%] in the retrospective cohort study) and the prevalence of microcephaly among newborns (5 [8.1%] in the prospective cohort study and 6 [7.3%] in the retrospective cohort study) were higher among newborns from women infected with *P falciparum* during pregnancy. Multivariate logistic regression analyses revealed that *P falciparum* infection during pregnancy represented a significant risk factor for the occurrence of small head circumference in newborns (prospective cohort study: odds ratio, 3.15; 95% CI, 1.52-6.53; *P* = .002; retrospective cohort study: odds ratio, 1.91; 95% CI, 1.21-3.04; *P* = .006). Placental pathologic findings corroborated this association, with more syncytial nuclear aggregates and inflammatory infiltrates occurring in placentas of newborns born with decreased head circumference.

**Conclusions and Relevance:**

This study indicates that falciparum malaria during pregnancy is associated with decreased head circumference in newborns, which is in turn associated with evidence of placental malaria.

## Key Points

**Question:**

Is malaria infection during pregnancy associated with fetal head growth?

**Findings:**

In 2 cohort studies of 4291 pregnancies, falciparum malaria during pregnancy was significantly associated with the occurrence of decreased head circumference in newborns. Placental malaria characterized by increased placental syncytial nuclear aggregates, leukocyte infiltration, and imbalanced angiogenic factors was associated with the incidence of decreased head circumference.

**Meaning:**

*Plasmodium falciparum* infection during pregnancy was associated with altered fetal head development, with possible consequences for fetal neurologic development.

## Teaser

This cohort study conducted in the Amazonian region of Brazil investigates whether an association exists between malaria infection during pregnancy and fetal head growth assessed by head circumference of newborns.

## Introduction

Having malaria during pregnancy, especially falciparum malaria, can be devastating and fulminant, leading to high mortality rates for both mother and fetus, with approximately 125 million pregnancies at risk of infection each year.^1^ Infected erythrocytes accumulate and sequester in the placental intervillous space, causing placental histopathologic changes that trigger an exacerbated inflammatory response that is highly detrimental.^2^ A heightened inflammatory response perturbs the maternal-fetal interface and impairs critical placental functions. Therefore, maternal malaria is associated with major complications among fetuses and newborns, including being the primary cause of low birth weight, intrauterine growth retardation, abortion, stillbirth, premature delivery, and fetal death in malaria-endemic countries.^1^ In addition, in utero exposure to malaria parasites has been associated with a decreased head circumference (HC) among fetuses and newborns, a proportional decrease in size as an outcome of the intrauterine growth retardation.^3,4,5,6^

Several studies have reported the association of intrauterine infections with a high risk of low birth weight and brain injury among newborns.^7^ A group of microorganisms termed *TORCH*, which now also includes hepatitis virus, HIV, and most recently Zika virus, are frequently associated with decreased HC in newborns.^8,9^Although the brain insult is defined by the cranium size, it also reflects a reduction of brain volume and an impairment of cognitive abilities.^10^ Thus, to investigate the association of having malaria during pregnancy with fetal head growth, we analyzed data from both a prospective and a retrospective cohort of newborns who were delivered between 2012 and 2015 in the far western Brazilian Amazonian region.

## Methods

### Study Site and Ethical Approval

Two cohort studies were conducted in the Amazonian region of the Alto do Juruá valley (Acre, Brazil) between January 2012 and April 2015, evaluating data from maternal-child pairs for births occurring in the general maternity ward at Hospital da Mulher e da Criança do Juruá (Cruzeiro do Sul), where approximately 90% of total deliveries in the region occur. The Alto do Juruá valley is in the extreme southwest of the Brazilian Amazon Basin, covering an area of 74 965 km^2^, predominantly rainforest, and containing a population of approximately 200 000 inhabitants (eFigure 1 in the Supplement). The region investigated in this study has an annual parasite incidence rating above 100. The annual parasite incidence represents the total number of malaria cases occurring yearly in a region per 1000 inhabitants; a rating equal to or greater than 50 is considered high-risk area for malaria transmission. *Plasmodium falciparum* is responsible for about 30% of malaria cases in the Acre state, which accounts for approximately 46% of the total *P falciparum* malaria Brazilian cases.^11,12^ In this region, 18% of women acquire *Plasmodium* infection during pregnancy.^13^ The present study was conducted in accordance with the Declaration of Helsinki^14^ and was registered in the Brazilian Clinical Trials Registry (RBR-3yrqfq). This article was written according to the Strengthening the Reporting of Observational Studies in Epidemiology (STROBE) reporting guideline for cohort studies. All authors agreed to maintain the confidentiality of the data collected from the medical records and databases by signing the Term of Commitment for the Use of Data from Medical Records. Ethical approval was provided by the committees for research of the University of São Paulo and the Federal University of Acre (Plataforma Brasil, CAA), according to the Brazilian National Health Committee. All study participants or their legal guardians (if minors) provided written informed consent.

### Prospective Cohort Study

In total, 600 pregnant women who were enrolled between January 2013 and April 2015 through volunteer sampling and who were infected with *P falciparum* or *Plasmodium vivax* or were noninfected were followed up until delivery. During this period, all pregnant women diagnosed as having malaria by the local endemic surveillance teams were invited to participate in the study. Women who were initially considered noninfected were recruited during their first visit to the antenatal care clinic. Each woman was followed up by a trained nurse, which involved at least 2 domiciliary visits, at the second and third trimester, to monitor the woman’s clinical state in addition to the usual antenatal care.

At the time of recruitment, data were collected on socioeconomic, clinical, and obstetric variables, and peripheral blood and thick and thin blood smears were used to diagnose and confirm malaria infection. During the domiciliary visits, clinical and obstetric data were obtained and a peripheral blood sample was collected. An additional blood sample was collected for each episode of malaria during pregnancy. At the time of delivery, clinical data were collected from the mother, newborn, a placental fragment, and blood samples.

The gestational age was estimated using the woman’s last menstrual period and adjusted using ultrasonographic data during the first trimester of pregnancy. Based on the gestational age, the HC, and sex, each newborn was assigned to groups using the definitions of the Intergrowth-21st Project.^15^ An individual was considered within normal reference head circumference (NHC) range if their HC was within 1 SD of the mean. Newborns with an HC less than 1 SD below the mean were considered to have a small head (SH),^16^ and newborns with an HC less than 2 SDs below the mean were classified as having microcephaly.^10^ Detailed procedures for data collection, sample processing, malaria screening and treatment, other infectious agent screening, angiogenic factors and leptin levels, and newborns’ anthropometric measurements are given in the eAppendix in the Supplement. The histopathologic examination involved using placental tissue slides, which were stained with hematoxylin-eosin; polarized light microscopy; and immunohistochemistry for different cell types. Additional details are given in the eAppendix and eTable 1 in the Supplement.

### Retrospective Cohort Study

In total, 4697 maternal-child pairs were selected retrospectively through a population-based sampling of all deliveries occurring between January 2012 and December 2013. The data from the Brazilian Epidemiological Surveillance Information System (SIVEP)–Malaria on maternal malaria infection status during pregnancy were assembled with the clinical and anthropometric data presented in the medical records of the mother and the newborn. This was followed by the collection and collation of data to evaluate the newborns further (details given in the eAppendix in the Supplement).

The gestational age was established by the woman’s last menstrual period, which was obtained from medical records. On the basis of the estimation of gestational age, HC, and sex, each newborn was assigned to groups using the World Health Organization child growth standards.^17^ These guidelines provide an appropriate reference standard for term *neonates* when gestational age is not acquired through ultrasonography.^18^ An individual was considered within the NHC range if the HC was within 1 SD of the median (boys, 33.2 cm ≤ HC ≤ 35.7 cm; girls, 32.7 cm ≤ HC ≤ 35.1 cm). Newborns with an HC less than 1 SD below the median were considered to have an SH (boys, HC <33.2 cm; girls, HC <32.7 cm).^16^ Newborns with an HC less than 2 SDs below the median were classified as having microcephaly (boys, HC <31.9 cm; girls, HC <31.5 cm).^17^ Detailed procedures on malaria and other infectious agent screening, malaria treatment, and newborn anthropometric measurements are given in the eAppendix in the Supplement.

### Exclusion Criteria

Our analysis was restricted to newborns who had been born at term (37-42 weeks of gestation) with at least 2500 g of weight, as a single birth, and from mothers of fertile age (13-47 years old). Women were excluded if they had a history during pregnancy of smoking, drug use, or alcohol consumption or presented with other infections (TORCH, HIV, hepatitis B virus, hepatitis C virus, syphilis, dengue, chikungunya, or Zika virus) or other comorbidities (hypertension, preeclampsia/eclampsia, diabetes, preterm delivery, abortion, stillbirth, and newborn with a congenital malformation) (eTable 4 and eTable 5 in the Supplement). Owing to the high percentage of cesarean deliveries performed in Brazilian maternity units, women who underwent cesarean delivery were not excluded from the study.

### Statistical Analysis

Data were analyzed from January to August 2017 and revised in November 2018 using R (The R Foundation), Stata (StataCorp), and GraphPad Prism software. Continuous variables were summarized using mean (SD) values as well as median values and interquartile ranges (IQRs). Categorical variables were summarized using frequencies and percentages. Differences between groups were evaluated using the nonparametric Kruskal-Wallis test followed by the Dunn post hoc multiple comparison test and the Mann-Whitney test as appropriate. Categorical data and proportions were analyzed using χ^2^ tests. All *P* values were 2 sided, and *P* < .05 was considered statistically significant. To assess the association between malaria and HC reduction, adjusted odds ratios (ORs) with 95% CIs were estimated using a multivariate logistic regression approach. These models included infection by malaria (no or yes), maternal age (≥18 or ≤17 years old), and the number of gestations (≥2 or 1) as explanatory variables and SH (yes or no) or microcephaly (yes or no) as response variables. The first category for each explanatory variable was considered the reference.^19^ Missing data were imputed or filled in within a multiple imputation framework using the MICE library within R software.^20,21^ In particular, in the retrospective cohort study, 5 data sets were completed, and the results were pooled across, allowing for the uncertainty in the imputation process.

## Results

### Study Population

**Figure 1.**
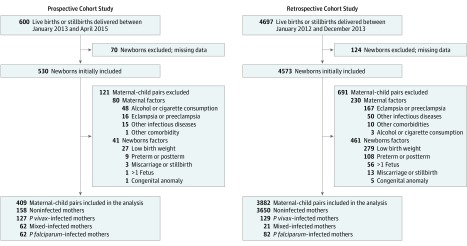
Flow Diagram of the 2 Cohort Studies Detailing Exclusion Criteria Mixed infection indicates that *Plasmodium vivax* and *Plasmodium falciparum* infections occurred at the same time or at different times during pregnancy.

In total, 600 pregnant women were enrolled in the prospective cohort study and followed up until delivery. Of the first eligible maternal-child pairs, 409 (68.2%) met the inclusion criteria (Figure 1). Among the 409 newborns, the mothers of 251 newborns had malaria during pregnancy, infected with *P vivax*, *P falciparum*, or both (mixed) (Figure 1). There were no relevant maternal or newborn baseline differences among the 4 distinct groups (Table 1).

**Table 1.** Baseline Characteristics of Mothers and Newborns in the Prospective Cohort Study

Characteristic	Noninfected (n = 158)	*Plasmodium vivax* (n = 127)	Mixed Infection (n = 62)^a^	*Plasmodium falciparum* (n = 62)
Mothers				
Maternal age, mean (SD), y	24.3 (6.2)	22.2 (6.2)	23.3 (5.8)	23.1 (6.3)
Gravidity, No. (%)				
Primigravida	72 (45.6)	51 (40.2)	19 (30.7)	23 (37.1)
Multigravida	86 (54.4)	76 (59.8)	43 (69.3)	39 (62.9)
Gestational age at delivery, wk				
Mean (SD)	39.7 (1.2)	39.6 (1.3)	39.5 (1.2)	39.6 (1.3)
Median (IQR)	40.0 (39.0-40.0)	40.0 (39.0-41.0)	40.0 (39.0-40.0)	39.5 (39.0-40.0)
Cesarean delivery, No. (%)	90 (57.0)	51 (40.2)	27 (43.6)	23 (37.1)
Weight gain, mean (SD), kg^b^	13.6 (5.0)	11.2 (5.0)	12.0 (5.0)	11.8 (5.2)
Hematocrit, mean (SD), %^c^	36.2 (3.5)	35.3 (3.9)	34.9 (4.0)	34.2 (4.3)
Hemoglobin, mean (SD), g/dL^d^	11.9 (1.2)	11.6 (1.3)	11.5 (1.3)	11.2 (1.4)
Placental weight, mean (SD), g^e^				
Primigravida	578.8 (97.0)	558.7 (102.2)	533.3 (63.1)	568.0 (129.2)
Multigravida	608.0 (112.2)	601.5 (148.9)	578.8 (108.8)	592.6 (159.1)
Antenatal care visits, mean (SD)^f^	7.9 (2.3)	6.4 (2.5)	6.3 (2.3)	5.6 (2.8)
Previous malaria episodes during current pregnancy, No. (%)	NA	37 (29.1)	30 (48.4)	7 (11.3)
Newborns				
Male newborns, No. (%)	72 (45.6)	68 (53.5)	35 (56.5)	29 (46.8)
Weight, g				
Male				
Mean (SD)	3244.6 (354.5)	3369.9 (384.7)	3268.2 (340.7)	3304.3 (402.2)
Median (IQR)	3250.0 (3012.5-3477.5)	3360.0 (3067.5-3625.0)	3250.0 (3015.0-3420.0)	3320.0 (3055.0-3540.0)
Female				
Mean (SD)	3364.1 (422.2)	3172.4 (386.9)	3164.4 (425.7)	3125.5 (275.5)
Median (IQR)	3365.0 (3045.0-3630.0)	3065.0 (2865.0-3460.0)	3060.0 (2890.0-3400.0)	3115.0 (2935.0-3260.0)
Length, cm^g^				
Male				
Mean (SD)	49.3 (1.4)	49.6 (1.8)	49.5 (1.8)	49.6 (1.9)
Median (IQR)	49.0 (48.0-50.0)	49.0 (49.0-50.0)	50.0 (48.0-50.0)	49.0 (48.0-50.0)
Female				
Mean (SD)	49.5 (1.5)	48.9 (1.6)	49.0 (1.8)	49.0 (1.6)
Median (IQR)	49.5 (49.0-50.0)	49.0 (48.0-50.0)	49.0 (48.0-50.0)	49.0 (48.0-50.0)
Rohrer index^g^^,^^h^				
Male				
Mean (SD)	2.7 (0.3)	2.8 (0.3)	2.7 (0.3)	2.7 (0.2)
Median (IQR)	2.7 (2.5-2.9)	2.7 (2.6-2.9)	2.7 (2.5-2.9)	2.7 (2.5-2.9)
Female				
Mean (SD)	2.8 (0.3)	2.7 (0.2)	2.7 (0.3)	2.7 (0.2)
Median (IQR)	2.8 (2.6-2.9)	2.7 (2.5-2.9)	2.7 (2.5-2.9)	2.7 (2.5-2.9)
Head circumference, cm				
Male				
Mean (SD)	34.4 (1.3)	34.4 (1.3)	34.4 (1.4)	33.8 (1.3)
Median (IQR)	34.0 (33.5-35.0)	34.0 (34.0-35.0)	34.0 (34.0-35.0)	34.0 (33.0-35.0)
Female				
Mean (SD)	34.3 (1.3)	33.8 (1.3)	33.7 (1.6)	33.5 (1.7)
Median (IQR)	34.0 (34.0-35.0)	34.0 (33.0-35.0)	34.0 (33.0-35.0)	34.0 (32.0-35.0)
Apgar Score^i^^,^^j^				
1 min				
Male				
Mean (SD)	8.2 (1.3)	8.4 (0.7)	8.1 (1.1)	8.4 (0.8)
Median (IQR)	9 (8-9)	9 (8-9)	8 (8-9)	9 (8-9)
Female				
Mean (SD)	8.4 (0.8)	8.4 (1.2)	8.4 (0.9)	8.4 (0.7)
Median (IQR)	9 (8-9)	9 (8-9)	9 (8-9)	8 (8-9)
5 min				
Male				
Mean (SD)	9.3 (0.8)	9.5 (0.5)	9.3 (0.7)	9.6 (0.6)
Median (IQR)	9 (9-10)	10 (9-10)	9 (9-10)	10 (9-10)
Female				
Mean (SD)	9.5 (0.5)	9.4 (0.6)	9.6 (0.5)	9.4 (0.6)
Median (IQR)	9 (9-10)	9 (9-10)	10 (9-10)	9 (9-10)

Abbreviations: IQR, interquartile range; NA, not applicable.

SI conversion factors: To convert hematocrit to proportion of 1.0, multiply by 0.01; hemoglobin to grams per liter, by 10.0.

^a^ Mixed infection: *P vivax* and *P falciparum* infection occurring at the same time or at different times during pregnancy.

^b^ Maternal weight gain (determined by subtracting the initial pregnancy weight from the final weight) was recorded in 153 noninfected and 107 *P vivax–,* 56 mixed-, and 49 *P falciparum*–infected pregnant women.

^c^ Hematocrit was recorded in 107 noninfected and 86 *P vivax–*, 43 mixed-, and 35 *P falciparum*–infected pregnant women.

^d^ Hemoglobin was recorded in 107 noninfected and 85 *P vivax–*, 43 mixed-, and 35 *P falciparum*–infected pregnant women.

^e^ Placental weight was recorded in 148 noninfected and 108 *P vivax–*, 57 mixed-, and 48 *P falciparum–*infected pregnant women.

^f^ The number of antenatal care visits was recorded in 153 noninfected and 120 *P vivax–*, 59 mixed-, and 57 *P falciparum–*infected pregnant women.

^g^ Length and Rohrer index were recorded in 157 newborns from noninfected pregnant women.

^h^ The Rohrer index is the newborns’ weight in grams divided by the cube of the length in centimeters, and newborns were considered proportional when values were between 2.32 and 2.85.

^i^ Apgar scores 7 to 10, normal reference range; 4 to 6, some breathing assistance might be required; and less than 4, more assistance must be provided.

^j^ Apgar score at 1 and 5 minutes was recorded in 153 newborns from noninfected and 112 *P vivax–*, 58 mixed-, and 52 *P falciparum–*infected pregnant women.

### Association of *P falciparum* Infection During Pregnancy With Newborn HC

**Figure 2.**
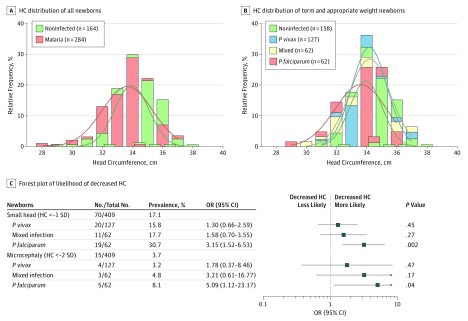
Association of Newborn Head Circumference (HC) With Malaria Infection During Pregnancy in the Prospective Cohort Study Newborn HC frequency distribution by maternal infection status. A, Mean (SD) HC for malaria-infected (33.71 [1.75] cm) and noninfected (34.19 [1.54] cm) mothers (*P* = .005). B, Mean (SD) HC for noninfected mothers (34.33 [1.29] cm) and mothers infected with *Plasmodium vivax* (34.13 [1.35] cm), *Plasmodium falciparum,*(33.61 [1.48] cm), or mixed *P vivax* and *P falciparum* occurring at the same time or at different times during pregnancy (34.10 [1.51] cm) after excluding low-birth-weight and preterm newborns (noninfected vs *P falciparum, P = *.02). Differences between each group were determined by Mann-Whitney rank sum tests (A) and Kruskal-Wallis tests with Dunn corrections (B). C, Forest plot of the odds ratios of small HC or microcephaly among newborns born from women infected during pregnancy by *Plasmodium* species compared with newborns from noninfected mothers. *P* values estimated through multivariate logistic regression methods.

The frequency distribution of the HCs among newborns showed differences between newborns from noninfected mothers and malaria-infected mothers, with the latter displaying a peak deviated from and spread to the left of the that for the noninfected mothers. This result is indicative of more newborns from malaria-infected mothers with decreased HC compared with those from noninfected mothers (mean [SD], 33.71 [1.75] cm vs 34.19 [1.54] cm; *P* = .005) (Figure 2A). To ensure that the observed difference was not associated with low birth weight (eTable 2 in the Supplement) or with preterm delivery, such newborns were removed from the analysis, and the malaria-infected group was separated into specific *Plasmodium* species–infected groups. Even with these changes, the peak of the HC distribution in the *P falciparum–*infected group (mean [SD], 33.61 [1.48] cm) deviated from that in the noninfected group (mean [SD], 34.33 [1.29] cm; *P* = .02) (Figure 2B), indicating a higher frequency of newborns with smaller HC among *P falciparum–*infected mothers.

Among 409 evaluated newborns in the prospective cohort study, 70 (17.1%) presented with an SH, including 15 (3.7%) with microcephaly (Figure 2C). The evaluated newborns were considered proportionate based on the Rohrer index, independent of the HC (eTable 3 in the Supplement). To evaluate the association of malaria during pregnancy with fetal head growth, the newborns were segregated by their HC and maternal infection status of noninfected, *P vivax* infection, mixed infection, or *P falciparum* infection. The prevalence of newborns with an SH was higher among newborns from women infected with *P falciparum* during pregnancy (19 [30.7%]). Similarly, the prevalence of microcephaly doubled when a *P falciparum* malaria infection occurred (5 [8.1%]) (Figure 2C). A multivariate logistic regression analysis revealed that *P falciparum* infection during pregnancy was associated with the likelihood of having an SH (OR, 3.15; 95% CI, 1.52-6.53; *P* = .002) or microcephaly (OR, 5.09; 95% CI, 1.12-23.17; *P* = .04) among the newborns (Figure 2C). Infection with *P vivax* during pregnancy was not found to be associated with decreased HC (for SH: OR, 1.30, 95% CI, 0.66-2.59; *P* = .45). None of the newborns included in the association analysis tested positive for TORCH infections, syphilis, HIV, dengue, chikungunya, or Zika virus (eTable 4 and eTable 5 in the Supplement).

### Association of Placental Malaria With Newborn HC

Several placental factors were evaluated to ascertain the association of placental malaria caused by *P falciparum* infection with the occurrence of an SH. Mothers whose newborns had an SH (*P falciparum*–associated SH) experienced their first *P falciparum* infection later in gestation (median, 25.5 weeks; IQR, 18.0-32.5 weeks) than mothers whose newborns had an NHC (median, 19.0 weeks; IQR, 12.0-29.3 weeks; *P* = .01). Moreover, much of the placental malaria manifestation in newborns with an SH (*P falciparum–*SH; 13 of 24 [54%]) or microcephaly (*P falciparum–*microcephaly; 5 of 7 [72%]) could be associated with a past *P falciparum* infection compared with that for *P falciparum*–associated NHC (38 of 80 [48%]) (Table 2).

**Table 2.** Infection Characteristics Among *Plasmodium falciparum*–Infected Pregnant Women Stratified by Head Circumference of Newborns in the Prospective Cohort Study

Characteristic	*P falciparum–*NHC Group (n = 94)	*P falciparum–*SH Group (n = 30)	*P* Value^a^	*P falciparum*–MC Group (n = 8)	*P* Value^b^
Infections per pregnancy, median (IQR)	2.0 (1.0-3.0)	2.0 (1.0-2.0)	.46	1.0 (1.0-2.0)	.12
Parasitemia of first infection, median (IQR)^c^	1.2 (0.3-4.6)	3.8 (0.5-9.2)	.05	0.4 (0.2-1.8)	.22
Gestational age at first infection, wk					
Mean (SD)	20.7 (10.5)	26.0 (8.1)	.01	27.6 (7.8)	.06
Median (IQR)	19.0 (12.0-29.3)	25.5 (18.0-32.5)		28.5 (19.8-34.3)	
Placental malaria, No. (%)^d^					
No	29 (36)	7 (30)	ND	1 (14)	ND
Active acute	8 (10)	2 (8)	ND	0	ND
Active chronic	5 (6)	2 (8)	ND	1 (14)	ND
Past	38 (48)	13 (54)	ND	5 (72)	ND
Hemozoin, No. (%)^d^^,^^e^					
No	31 (39)	8 (33)	ND	1 (14)	ND
Mild	32 (40)	9 (38)	ND	4 (57)	ND
Moderate	15 (19)	7 (29)	ND	2 (29)	ND
Severe	2 (2)	0	ND	0	ND

Abbreviations: IQR, interquartile range; MC, microcephaly; ND, not determined; NHC, normal head circumference; SH, small head.

^a^ Differences between *P falciparum–*NHC and *P falciparum–*SH groups were evaluated using Mann-Whitney rank sum tests.

^b^ Differences between *P falciparum–*NHC and *P falciparum–*MC groups were evaluated using Mann-Whitney rank sum tests.

^c^ Parasitemia was recorded in 82 *P falciparum–*NHC, 28 *P falciparum–*SH, and 7 *P falciparum–*MC cases. Values presented in 10^3^ DNA copies as obtained by photo-induced electron transfer–polymerase chain reaction quantification.

^d^ Placental malaria and hemozoin were recorded in 80 *P falciparum–*NHC, 24 *P falciparum–*SH, and 7 *P falciparum–*MC cases.

^e^ Mild, focal presence in small amounts; moderate, small spots or larger deposits in many locations; and severe, large amounts present widely.

**Figure 3.**
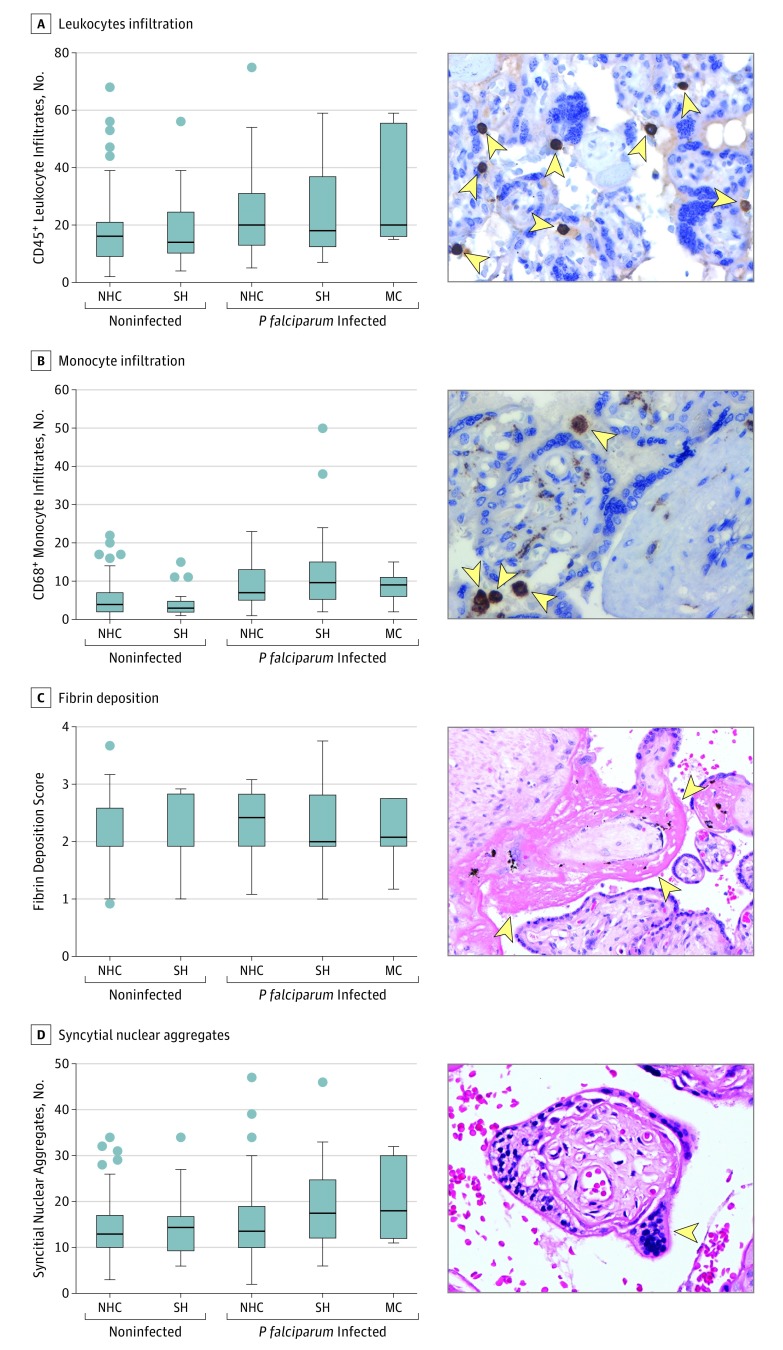
Evaluation of Histopathologic Factors of Placentas From Noninfected and *Plasmodium falciparum*–Infected Mothers by Newborn Head Circumference A, Number of CD45-positive (CD45^+^) leukocytes. B, Number of CD68-positive (CD68^+^) monocytes. C, Fibrin deposition score. D, Number of syncytial nuclear aggregates. Histopathologic factors were evaluated by microscopy using hematoxylin-eosin staining for fibrin deposition and syncytial nuclear aggregates and by immunohistochemistry techniques for leukocyte and monocyte infiltration. The photomicrographs were originally acquired at a magnification of ×40 (leukocytes and monocytes) and ×10 (fibrin deposits and syncytial nuclear aggregates). Because they are merely representative, they were further digitally magnified to better identify the pathology (arrowheads indicate the moiety analyzed in that row). Noninfected (n = 126-128); noninfected small head (SH) (n = 20); *P falciparum*–infected “normal” head circumference (NHC) (n = 54-80); *P falciparum–*SH (n = 17-24); and *P falciparum*–infected microcephaly (MC) (n = 5-7). Data are represented as Tukey boxplots, with the bottom and the top of the box representing the first and third quartiles; the band inside the box, the median; the whiskers, the lowest and the highest data points within 1.5 × the interquartile ranges of the first and upper quartiles; and the circles, outliers. Group differences were evaluated by Kruskal-Wallis tests with Dunn corrections.

The analysis of placental histologic and angiogenic factors disclosed substantial differences between noninfected controls and *P falciparum–*infected groups. Of note, compared with that for the noninfected group, we observed higher median numbers of infiltrated monocytes for all *P falciparum–*infected groups (*P falciparum–*NHC group, 7.0; IQR, 5.0-13.0; *P* < .001; *P falciparum–*SH group, 9.5; IQR, 5.5-15.0; *P* < .001; *P falciparum–*microcephaly group, 9.0; IQR, 6.0-11.0; *P* = .02 vs noninfected, 4.0; IQR, 2.0-7.0) (Figure 3B). However, syncytial nuclear aggregate (SNA) alterations were observed only in infected placentas of newborns with an SH or microcephaly. Syncytial nuclear aggregates presented excessive formation in the noninfected group (13.0; IQR, 10.0-17.0), in the *P falciparum–*SH group (17.5; IQR, 12.0-24.5; *P* = .01) and the *P falciparum–*microcephaly group (18.0; IQR, 12.0-30.0; *P* = .02) (Figure 3D). Moreover, the leptin levels (ng/mL) among the *P falciparum–*SH and *P falciparum–*microcephaly groups were lower than those in the noninfected group, although these decreases were not significant. (eFigure 2I in the Supplement). Details of the data can be found in eTable 6 in the Supplement. These results support placental dysfunction associated with *P falciparum* infection, with some factors, such as SNAs, being heightened in placentas derived from newborns with decreased HC.

### Association of *P falciparum* Infection With Newborn HC in Retrospective Cohort Study

In the population-based retrospective cohort study, 4697 maternal-child pairs were included. After application of the exclusion criteria, 3882 newborns (83%) remained to be evaluated, of which 232 were born from mothers who had malaria during pregnancy (Figure 1). Overall, there were no significant differences in baseline characteristics between the prospective cohort study and the retrospective cohort study (Table 1; eTable 7 in the Supplement). The evaluation of the frequency distribution of newborn HCs showed differences between newborns born from noninfected (mean [SD], 33.92 [1.76] cm) and malaria-infected mothers (mean [SD], 33.67 [1.60] cm; *P* = .008) (eFigure 3A in the Supplement). Identical to the prospective cohort study, when low-birth-weight newborns (eTable 2 in the Supplement) and preterm newborns were removed from the analysis and the malaria-infected group was segregated by infection status, the *P falciparum–*infected group presented a peak that deviated from that of the noninfected group (33.67 [1.49] cm vs 34.12 [1.55] cm, respectively; *P* = .04) (eFigure 3B in the Supplement). This finding is indicative of a higher frequency of newborns with decreased HC born to mothers who were infected with *P falciparum* during pregnancy.

Of 3882 evaluated newborns, 934 (24.1%) had an SH and 161 (4.2%) had microcephaly. In the retrospective cohort study, similar to the prospective cohort study, the prevalence of newborns with an SH was more than one-half times as high (36.6%), and microcephaly was doubled (7.3%), among newborns with *P falciparum–*infected mothers (eFigure 3C in the Supplement). Consistent with this finding, a multivariate logistic regression analysis revealed that *P falciparum* infection was associated with the odds of an SH occurring in newborns (OR, 1.91; 95% CI, 1.21-3.04; *P* = .006) (eFigure 3C in the Supplement). Taken together, these results showed that *P falciparum* infection during pregnancy was associated with the likelihood of decreased HC among newborns, supporting the results obtained in our prospective cohort study.

## Discussion

Malaria during pregnancy increases the risk of adverse fetal outcomes. This study shows evidence that infection with *P falciparum* during pregnancy is significantly associated with the occurrence of decreased HC in newborns and, to some extent, with microcephaly. The finding on the HC decrease is independent of the currently known association between malaria with overall fetal growth because low-birth-weight and preterm newborns were deliberately excluded from our analysis. An increased risk for decreased HC associated with *P falciparum* infection was supported by both our prospective cohort study (OR, 3.15; *P* = .002) and our retrospective cohort study (OR, 1.91; *P* = .006). These observations reinforce the knowledge that having malaria during pregnancy increases the risk of problems in fetal development.^1,2,22^

We hypothesize that the placental inflammatory process acting against the *P falciparum* infection may be a mechanism contributing to the decreased fetal head growth. This hypothesis was supported by the observed histopathologic alterations combined with an imbalance in angiogenic factor production in placentas from newborns with congenital SH or microcephaly born to *P falciparum–*infected mothers. Local inflammation can generate a setting of hypoxia or ischemia that would alter the transportation of both nutrients and respiratory gases to the unborn fetus, which can result in cranial malformation owing to an inadequate supply of nutrients and oxygen.^23^ Oxidative stress caused by hypoxia also leads to structural and functional alterations in intrauterine development.^24^ This scenario is often observed in cases of placental malfunction due to various etiologies and to prolonged or premature labor.^25^

The values of SNA, which have been associated with intrauterine growth retardation caused by local hypoxia or oxidative stress,^26^ were markedly increased in placentas from newborns with decreased HC. In addition, SNA has been repeatedly observed in placentas from *P falciparum–*exposed women.^2,27,28^ The placental alterations observed in this study, including the increased SNA and monocyte infiltration, are consistent with previous reports on the response to placental *P falciparum–*sequestering parasites, which characterizes placental malaria development.^2,22,28^ It was unsurprising that infection with *P vivax* was not associated with a decreased HC phenotype because this parasite is known to not sequester in the placenta. Previous studies have demonstrated that *P vivax* infection induces less of a placental inflammatory process compared with that induced by *P falciparum* infection.^28^

Regarding the *P falciparum–*SH group, few observed differences reached statistical significance in this study, possibly owing to the small sample size of this group, but the overall placental malaria phenotype was more prominent and widespread than that in the noninfected and *P falciparum–*NHC groups. Nevertheless, our observations reflect only a picture at the moment of birth, and it is unclear how placental alterations may be associated with the development of the fetus.

Currently, much of what is known about falciparum gestational malaria is based on studies performed in high-transmission areas in Africa, which, in general, are settings that have precarious health systems and inadequate or late treatment provision. In Brazil, approximately 85% of malaria infections are caused by *P vivax.* However, *P falciparum* is transmitted in specific regions, including the Alto do Juruá valley, where it is responsible for 46% of the total malaria infections in Brazil.^11,13^ Despite Brazil being an area with low transmission for malaria, effective control strategies, and early treatment provision, we observed adverse events in newborns similar to those reported in areas of high endemicity.

Surprisingly, the prevalence of microcephaly observed by us is far higher than what has been previously reported by the Brazilian Ministry of Health.^10^ Recently, other studies that have evaluated newborns retrospectively in different Brazilian regions have also reported a higher prevalence of microcephaly in newborns before the Zika outbreak.^29,30^ It is puzzling that the United States, with nearly the same number of births per year as Brazil, reports approximately 25 000 newborns with microcephaly yearly, whereas Brazil reported only 150 before the Zika epidemy.^31,32^ These observations indicate an inconsistency of the data released by the Brazilian authorities, likely owing to underreporting.

### Limitations

Our work has some potential limitations. First, the HC among newborns was assessed only at birth because morphometric measurements through ultrasonography during pregnancy, as well as the possibility of acquiring newborn head imaging, were not possible. Second, decreased HC has different etiologies, namely, genetic causes and actions of infectious agents. Although we removed some confounding factors, such as TORCH infections, syphilis, HIV, dengue, chikungunya, Zika virus, and other comorbidities, studies to detect genetic abnormalities among the patients were not performed. Third, although in both the prospective cohort study and the retrospective cohort study the logistic regression analyses indicated a clear association between an SH and *P falciparum* infection, we had access to only a few placentas. This small sample size limited our statistical analysis; however, most of the factors analyzed indicated intensified placental malaria compared with placentas from newborns with an HC within the normal reference range.

## Conclusions

This work provided evidence that *P falciparum* infection during pregnancy was associated with decreased HC and, in extreme cases, with microcephaly. The consequences of gestational malaria throughout fetal neurologic development, which can lead to poor neurocognitive and behavioral development, represent serious long-term health problems. Physicians should periodically assess the development and academic achievements of these children, with a comprehensive neurocognitive evaluation, to guide preventive and rehabilitative assistance that might improve outcomes. Extensive epidemiologic prospective studies, involving the collection of biological, clinical, and socioeconomic data and potential confounding factors, are required to establish the prevalence of newborns with an SH and the prevalence of newborns with microcephaly and their association with malaria. The evidence of the association observed in our study supports an urgent need to protect pregnant women and their unborn children from malaria infection.

## Notes

Supplement. **eAppendix.** Methods **eTable 1.** Evaluation Methods and Staining Used to Quantify Malaria-Associated Placental Parameters **eTable 2.** Baseline Characteristics of Low-Birth-Weight Newborns and Mothers of the Prospective and Retrospective Cohort Studies **eTable 3.** Baseline Characteristics of Newborns at Delivery of Non-Infected and *P. Falciparum*-Infected Pregnant Women of the Prospective Cohort Study **eTable 4.** Summary of the Serological Screening of TORCH, HIV, Syphilis, Dengue, Chikungunya and Zika Infections **eTable 5.** Results of the Screening of Other Infectious Agents in Mothers of Newborns With Small Head in the Prospective Cohort **eTable 6.** Placental Histological Parameters and Angiogenic Factors of Non-Infected And *P. Falciparum*-Infected Pregnant Women of the Prospective Cohort Study **eTable 7.** Baseline Characteristics of Mothers and Newborns of the Retrospective Cohort Study **eFigure 1.** Map Showing the Location of the Field Site, Alto do Juruá Region, Northwest of the Acre State, Brazilian Amazon **eFigure 2.** Placental Plasma Levels of Angiogenic Factors and Leptin From Non- and *P. Falciparum*-Infected Mothers According to Newborns Head Circumference **eFigure 3.** Retrospective Cohort Study Supports That Malaria Infection During Pregnancy Impacts Newborns Head Circumference **eReferences**
