# Supplementary material for: Association of Malaria Infection During Pregnancy With Head Circumference of Newborns in the Brazilian Amazon
Source: JAMA Netw Open. 2019 May 3;2(5):e193300. doi: 10.1001/jamanetworkopen.2019.3300 (PMC6503507; doi:10.1001/jamanetworkopen.2019.3300)
Supplement: Supplement. — eAppendix. Methods eTable 1. Evaluation Methods and Staining Used to Quantify Malaria-Associated Placental Parameters eTable 2. Baseline Characteristics of Low-Birth-Weight Newborns and Mothers of the Prospective and Retrospective Cohort Studies eTable 3. Baseline Characteristics of Newborns at Delivery of Non-Infected and P. Falciparum-Infected Pregnant Women of the Prospective Cohort Study eTable 4. Summary of the Serological Screening of TORCH, HIV, Syphilis, Dengue, Chikungunya and Zika Infections eTable 5. Results of the Screening of Other Infectious Agents in Mothers of Newborns With Small Head in the Prospective Cohort eTable 6. Placental Histological Parameters and Angiogenic Factors of Non-Infected And P. Falciparum-Infected Pregnant Women of the Prospective Cohort Study eTable 7. Baseline Characteristics of Mothers and Newborns of the Retrospective Cohort Study eFigure 1. Map Showing the Location of the Field Site, Alto do Juruá Region, Northwest of the Acre State, Brazilian Amazon eFigure 2. Placental Plasma Levels of Angiogenic Factors and Leptin From Non- and P. Falciparum-Infected Mothers According to Newborns Head Circumference eFigure 3. Retrospective Cohort Study Supports That Malaria Infection During Pregnancy Impacts Newborns Head Circumference eReferences [file jamanetwopen-2-e193300-s001.pdf]

Supplementary Online Content

Dombrowski JG, Souza RMd, Lima FA, et al. Association of malaria infection during pregnancy with head circumference of newborns in the Brazilian Amazon. *JAMA Netw Open*. 2019;2(5):e193300. doi: 10.1001/jamanetworkopen.2019.3300

eAppendix. Methods

eTable 1. Evaluation Methods and Staining Used to Quantify Malaria-Associated Placental Parameters

eTable 2. Baseline Characteristics of Low-Birth-Weight Newborns and Mothers of the Prospective and Retrospective Cohort Studies

eTable 3. Baseline Characteristics of Newborns at Delivery of Non-Infected and *P. Falciparum*-Infected Pregnant Women of the Prospective Cohort Study

eTable 4. Summary of the Serological Screening of TORCH, HIV, Syphilis, Dengue, Chikungunya and Zika Infections

eTable 5. Results of the Screening of Other Infectious Agents in Mothers of Newborns With Small Head in the Prospective Cohort

eTable 6. Placental Histological Parameters and Angiogenic Factors of Non-Infected And *P. Falciparum*-Infected Pregnant Women of the Prospective Cohort Study

eTable 7. Baseline Characteristics of Mothers and Newborns of the Retrospective Cohort Study

eFigure 1. Map Showing the Location of the Field Site, Alto do Juruá Region, Northwest of the Acre State, Brazilian Amazon

eFigure 2. Placental Plasma Levels of Angiogenic Factors and Leptin From Non- and *P. Falciparum*-Infected Mothers According to Newborns Head Circumference

eFigure 3. Retrospective Cohort Study Supports That Malaria Infection During Pregnancy Impacts Newborns Head Circumference

eReferences

This supplementary material has been provided by the authors to give readers additional information about their work.

eAppendix: Methods

Prospective Cohort Study - Samples processing

The peripheral and placental blood was collected in heparin tubes and then separated into plasma and whole blood cells using a centrifuge. Thin and thick blood smears were stained with Giemsa. The placental fragment was fixed in 10% neutral buffered formalin at 4°C until they could be sent to the University of São Paulo for processing. Paraffin-embedded 5µm sections of placental tissue were stained with Hematoxylin-Eosin (H&E) or Giemsa for histological examination. Total DNA was obtained from whole blood cells using a commercially available extraction kit (QIAmp DNA Mini Kit, Qiagen), following the manufacturer's instructions.

Prospective Cohort Study - Screening of malaria infection

Malaria during pregnancy was diagnosed from thin and thick blood smears by two experts in microscopy of the endemic surveillance team of Cruzeiro do Sul (Acre, Brazil). Furthermore, all samples collected throughout the pregnancy were screened for the presence of malaria parasites, by microscopy and real-time PCR technique (PET-PCR). This technique detects in multiplex the *Plasmodium* spp. and *P. falciparum*, and in singleplex *P. vivax* if only *Plasmodium* spp. is detected in the first PCR. PET-PCR has a detection limit of 3.2 parasites/µl.[1] The real-time PCR was performed on the 7500 Fast Real-Time PCR System (Applied Biosystems, ThermoFisher). All women who had malaria during pregnancy were treated with antimalarial drugs under medical prescription, according to the Brazilian Ministry of Health (MoH) guidelines,[2] with further treatment confirmation.

Prospective Cohort Study - Histopathology evaluation

The histopathologic examination involved using placental tissue slides. The Hematoxylin-Eosin-staining allowed evaluating the syncytial nuclear aggregates (SNA), fibrinoid necrosis, and fibrin deposition.[3] The hemozoin presence was assessed through microscopy of polarized light.[4] The leukocyte (CD45) and monocyte infiltrate (CD68), and the villous vascularity (CD31) have been evaluated by immunohistochemistry using the tissue microarray (TMA) technique, conducted at the AC Camargo Hospital, in São Paulo, Brazil, as described elsewhere.[5,6] The proliferation index was calculated through quantitative image analysis of anti-Ki-67/DAB staining.[7] All measurements were performed by two researchers blinded to group and outcome. Cases that proved to be contradictory between observers were re-evaluated until consensus was reached. eTable 1 describes these procedures in detail. Images were captured by a Zeiss Axio Imager M2 light microscope equipped with a Zeiss Axio Cam HRc camera and analyzed by Image J software (http://imagej.nih.gov/ij).

Prospective Cohort Study - Angiogenic factors and Leptin measurement

The angiogenic factors, vascular endothelial growth factor A (VEGFA, and it receptors VEGFR1/FLT1 and VEGFR2/FLK1), angiopoietins 1 and 2 (ANG-1 and ANG-2, and their associated soluble receptor the TEK receptor tyrosine kinase (TIE-2)), and the leptin hormone were measured in placental plasma using the DuoSet ELISA development kits (R&D), according to manufacturer's guidelines.

Prospective Cohort Study - Screening of other infectious agents

All pregnant women were screened in the local ANC clinics for toxoplasmosis, hepatitis, syphilis, and HIV by measuring antibodies titers, following the Brazilian MoH guidelines. Further, peripheral plasma from women that delivered newborns with small head and microcephaly, irrespective *of the infection status and Plasmodium species*, was tested to confirm the absence of other infectious agents during pregnancy. Tests for *Toxoplasma gondii*, Rubella, Cytomegalovirus, Herpes simplex virus, Syphilis, HIV, Dengue virus, Chikungunya virus, and Zika virus were performed retrospectively by ELISA assays in peripheral blood collected until the 28 weeks of gestation. In pregnant women that delivered newborns with microcephaly, plasma samples of two different time points of the pregnancy were tested. All the serological tests were performed using commercially available kits: HIV 1/2 and total Syphilis (Symbiosys) and IgG/IgM to Toxoplasmosis, Rubella, Cytomegalovirus and Herpes simplex (TORCH) (Virion\Serion), and used according to the manufacturer's instructions. To detect Dengue, Chikungunya, and Zika current viral infections, qualitative assays were carried out by IgM capture using a specific viral antigen for DENV, ZIKV, and CHIKV, as previously described.[8] The identification of specific IgG antibodies to CHIKV was performed using a specific viral antigen,[8] and to DENV and ZIKV were made with an antigen derived from a whole DENV-2 NS1 protein and a portion of the NS1 protein, respectively (unpublished data). Developing color was quantified on an automatic microliter plate reader Spectramax Plus 384 (Molecular Devices). The results were expressed as optical density (OD) at 405/630 nm or 450/630 nm (Virion/Serion and Symbiosys/Alka Kits, respectively). In TORCH analyses, the presence of IgG and IgM antibodies were classified as positive, negative or borderline according to an OD range adopted by standard positive control mean. For Rubella and *Toxoplasma gondii* (IgG) avidity test was performed according to the manufacturer's specifications (Virion/Serion), and in all TORCH IgM tests, we use the rheumatoid factor absorbent reagent (# Z200, Virion/Serion). All the kits followed the validation criteria, and the presence of IgG and IgM antibodies for Syphilis and HIV antigens were determined by comparing the absorbance

© 2019 Dombrowski JG et al. *JAMA Network Open*.

value of serum samples with the cut-off value of standards of reference controls and classified as positive or negative. All tests were performed without the operator knowledge of the group classification for each sample. If the test was inconclusive, the screening was repeated using samples from two different gestational time-points. Newborns were excluded from the analysis whenever their mothers presented antibody titers for IgM.

Prospective Cohort Study – Deviation from the Original Plan
The current sample sizes present a deviation from those proposed at the outset. It was proposed to enroll ~400 infected and ~800 non-infected pregnant women into the prospective cohort study. We were unable to recruit the 2:1 ratio, as some initially included in the non-infected group, were transferred to an infected group upon *Plasmodium* molecular detection.

Retrospective Cohort Study – Retrospective record review
The retrospective record review was performed based on the question "Does malaria during pregnancy in "Alto do Juruá" impacts newborns morphometrics?". Therefore, data on clinical information about births and maternal malaria were linked. The medical records of all consecutive births occurred in the Juruá Women's and Children's Hospital (Cruzeiro do Sul, Acre - Brazil), between January 2012 and December 2013 were used. Since the medical records were paper-based, it was initially digitalized, and further all data was extracted and introduced in a computerized database. The information on the maternal malaria episodes and parasite species, between 2011 and 2013, was obtained from the Brazilian Epidemiological Surveillance Information System (SIVEP)-Malaria, as a computerized database. The Secretaries of Health from the State of Acre and the city of Cruzeiro do Sul granted the consent to use these datasets after signing the Term of Commitment for the Use of Data from Medical Records under the agreement of maintaining confidentiality and safety of the collected data from the medical records and databases. Before performing the record linkage, curation was achieved, and newborns with double entries, lack of information on head circumference size and on maternal name and age were excluded. Standardization of both databases was performed by withdrawing accentuations, extra spaces, special characters, and prepositions. The record linkage of the two databases was performed only by two shared variables that presented the appropriate fulfillment, the maternal name, and age. The name variable was strictly used for the record linkage, which was removed, and data anonymized before data analysis. The data abstraction from the linked database was performed based on unambiguous variables and inclusion, and exclusion criteria established *a priori* and used in our analyses. Two independent and trained researchers performed all this procedure.

Retrospective Cohort Study - Screening of malaria infection
Malaria during pregnancy was diagnosed from thin and thick blood smears by microscopists of the endemic surveillance team of Cruzeiro do Sul (Acre, Brazil), whenever women show suspicious malaria symptoms. These data were obtained from the Brazilian Epidemiological Surveillance Information System (SIVEP)-Malaria. In Brazil, malaria is a compulsory notification disease, with all the suspected or confirmed cases mandatorily notified to the health authorities by SIVEP-Malaria. This system covers all states of the Amazon region, where more than 95% of malaria cases occur. All women who had malaria during pregnancy were treated with antimalarial drugs under medical prescription, according to the Brazilian MoH guidelines [2].

Retrospective Cohort Study - Screening of other infectious agents
All pregnant women were screened in the local ANC clinics for toxoplasmosis, hepatitis, syphilis, and HIV by measuring antibodies titers, following the Brazilian MoH guidelines.

Prospective and Retrospective Cohort Studies - Malaria treatment
All women who had malaria during pregnancy were treated with antimalarial drugs under medical prescription, according to the Brazilian Ministry of Health (MoH) guidelines.[2] In short, for a non-complicated *P. vivax*-malaria infection in pregnant women, it is recommended the use of Chloroquine in a three-day treatment schedule. For infections with *P. falciparum,* during the first trimester it is advocated the use of Quinine for seven days in association with clindamycin for five days; for the second and third gestational trimesters, it is recommended the use of Artemether and Lumefantrine in a three-day schedule, in fixed-combination. In Brazil, it is not prescribed the prophylaxis through the intermittent preventive treatment with Sulphadoxine-Pyrimethamine (IPTp-SP).[2]

Prospective and Retrospective Cohort Studies - Newborn anthropometric measures
In the two cohort studies, PCS and RCS, the newborn's anthropometric measures were obtained within 24h of the delivery, by trained nurses. Weight was measured in grams (g) using digital pediatric scales, with a precision of 5 g, and the length and occipitofrontal HC were measured in centimeters (cm), using a non-stretching flexible measuring tape. Rohrer's ponderal index is the newborns' weight in grams divided by the cube of the length in centimeters, and newborns are considered proportional when values are between 2.32 and 2.85, corresponding to the 10th percentile.[9,10]

© 2019 Dombrowski JG et al. *JAMA Network Open*.

An Apgar score indicates the physical condition of the newborn, relative to its response to stimulation, skin coloration, heart rate, respiratory effort, and muscle tone.[11]

eTable 1. Evaluation Methods and Staining Used to Quantify Malaria-Associated Placental Parameters

	Evaluation methods	Staining	Company
**Pathological features**			
Syncytial nuclear aggregates	Number of affected villi per 100 villi at 10× magnification.[3,12]	Hematoxylin-Eosin	-
Fibrin deposition score	Semi-quantitative scoring was used for placental fibrin on a scale from 0 to 5. For the extent of fibrin deposition at the basal and chorionic plates and intervillous fibrin and perivillous fibrin, the following scale was used to apply a score to each: none (0), scant (1), minimal extension (2), moderate (3), heavy (4), or extensive (5) at 100× magnification.[3,12]	Hematoxylin-Eosin	-
Fibrinoid Necrosis	Number of intersection points on a random grid that touched areas of necrosis per total points of a square grid 4 862.43 μm^2 of area point at 10× magnification.[3,12]	Hematoxylin-Eosin	-
Proliferation index (Ki-67)	The index was calculated by quantitative image analysis of the percentage of positively stained nuclear area with anti-Ki-67/DAB per the total area of the nuclei, obtained by averaging three images of the same sample at the 20-fold increase. Employing a web available free application for ImageJ.[7]<br>Clone: SP6 - Dilution 1:1000	Immunohistochemistry	*Spring* (Pleasanton, EUA)
Villous vascularity ($CD31^+$)	Number of fetal vessels labeled with anti-CD31 in ten villi terminals at 20× magnification in the Axio Scan.Z1 scanning system.<br>Clone: JC70 - Dilution 1:2000	Immunohistochemistry	*Cell Marque* (Rocklin, EUA)
Leukocytes infiltrate ($CD45^+$)	Number of leukocyte cells in 10 fields at 400× magnification.<br>Clone: 2B11 GDP7/26 - Dilution 1:4000	Immunohistochemistry	*Cell Marque* (Rocklin, EUA)
Monocytes infiltrate ($CD68^+$)	Number of monocyte cells in 10 fields at 400× magnification.[5,6,13]<br>Clone: KP1 - Dilution 1:1000	Immunohistochemistry	*Cell Marque* (Rocklin, EUA)
**Malaria-associated features**			
Parasitized erythrocytes	Number of fields with the parasite in 100 fields at 1000× magnification.	Giemsa	-
Hemozoin	Sixty fields at 40× magnification were screened with polarized light for the presence of hemozoin in the intervillous space (free or within cells) and in the tissue.[4]	Hematoxylin-Eosin	-

© 2019 Dombrowski JG et al. *JAMA Network Open*.

eTable 2. Baseline Characteristics of Low-Birth-Weight Newborns and Mothers of the Prospective and Retrospective Cohort Studies.

Characteristics	Prospective Cohort Study				Retrospective Cohort Study			
	Non-Infected (N=5)	*P. vivax* (N=14)	Mixed (N=4)	*P. falciparum* (N=4)	Non-Infected (N=245)	*P. vivax* (N=21)	Mixed (N=2)	*P. falciparum* (N=11)
**Mothers**								
Maternal age, mean (SD), y[a]	24.4 (7.1)	22.3 (6.3)	18.8 (6.3)	15.8 (0.9)	23.3 (7.2)	22.1 (6.1)	20.0 (2.8)	20.4 (4.3)
Gravidity, No. (%)[b]								
Primigravida	4 (80.0)	8 (57.1)	3 (75.0)	4 (100)	118 (50.2)	7 (33.3)	1 (50.0)	5 (62.5)
Multigravida	1 (20.0)	6 (42.9)	1 (25.0)	0	117 (49.8)	14 (66.7)	1 (50.0)	3 (37.5)
Gestational age at delivery, w								
Mean (SD)	36.4 (2.3)	37.0 (3.0)	33.8 (4.2)	33.5 (5.5)	36.5 (3.0)	37.1 (4.0)	35.5 (0.7)	37.8 (1.9)
Median (IQR)	38.0 (35.0-38.0)	37.0 (36.0-40.0)	34.5 (31.0-36.5)	33.5 (29.5-37.5)	37.0 (36.0-38.0)	38.0 (36.0-39.0)	35.5 (35.0-36.0)	38.0 (36.0-40.0)
C-section, No. (%)	3 (60.0)	7 (50.0)	0	0	108 (44.1)	11 (52.4)	0	4 (36.4)
Weight gain, mean (SD), Kg[c]	9.8 (3.3)	10.9 (4.6)	8.0 (2.8)	5.0 (1.2)	-	-	-	-
Hematocrit, mean (SD), %[d]	36.1 (5.5)	33.9 (3.4)	31.5 (5.1)	39.6 (2.3)	-	-	-	-
Hemoglobin, mean (SD), g/dL[e]	11.9 (1.6)	11.2 (1.1)	10.4 (1.6)	13.3 (0.9)	-	-	-	-
Placental weight, mean (SD), g[f]					-	-	-	-
Primigravida	456.4 (10.5)	388.6 (83.4)	422.0 (57.9)	413.7 (61.4)	-	-	-	-
Multigravida	-	436.4 (61.5)	461.8	-	-	-	-	-
Antenatal care visits, mean (SD)[g]	7.8 (2.8)	6.4 (2.4)	1.8 (1.0)	4.0 (1.4)	4.9 (2.4)	5.8 (1.7)	6.0 (4.2)	4.9 (2.7)
**Newborns**								
Male newborns, No. (%)	1 (20.0)	7 (50.0)	3 (75.0)	1 (25.0)	109 (44.5)	7 (33.3)	0	6 (54.6)
Weight, median (IQR), g								
Male	1945.0	2405.0 (2150.0-2480.0)	2120.0 (2070.0-2240.0)	2365.0	2240.0 (1920.0-2435.0)	2430.0 (2405.0-2480.0)	-	2400.0 (2270.0-2425.0)
Female	2400.0 (2072.5-2475.0)	2300.0 (1900.0-2370.0)	1950.0	2385.0 (1530.0-2415.0)	2290.0 (2110.0-2420.0)	2322.5 (2185.0-2370.0)	2252.5 (2155.0-2350.0)	2250.0 (2240.0-2290.0)
Length, median (IQR), cm[h]								
Male	45.0	46.0 (43.0-48.0)	45.0 (45.0-47.0)	51.0	45.0 (44.0-47.0)	46.0 (44.0-48.0)	-	47.0 (46.0-48.0)
Female	44.0 (41.5-46.0)	45.0 (42.0-47.0)	44.0	46.0 (43.0-47.0)	45.0 (43.0-47.0)	46.0 (44.0-47.0)	44.0 (44.0-44.0)	47.0 (45.0-49.0)

© 2019 Dombrowski JG et al. *JAMA Network Open*.

eTable 2. Baseline characteristics of low birth weight newborns and mothers of the Prospective and Retrospective Cohort Studies (continued)

Characteristics	Prospective Cohort Study				Retrospective Cohort Study			
	Non-Infected (N=5)	*P. vivax* (N=14)	Mixed (N=4)	*P. falciparum* (N=4)	Non-Infected (N=245)	*P. vivax* (N=21)	Mixed (N=2)	*P. falciparum* (N=11)
Rohrer index, median (IQR)[h,i]								
Male	2.13	2.36 (2.24-2.55)	2.27 (2.16-2.33)	1.78	2.36 (2.13-2.55)	2.50 (2.24-2.87)	-	2.23 (2.19-2.34)
Female	2.55 (2.34-3.18)	2.29 (2.22-2.56)	2.29	2.30 (1.92-2.48)	2.41 (2.24-2.66)	2.25 (2.22-2.58)	2.64 (2.53-2.76)	2.14 (2.09-2.17)
Head circumference, median (IQR), cm								
Male	30.0	33.0 (30.0-34.0)	29.0 (28.0-33.0)	33.0	31.0 (30.0-32.0)	33.0 (30.0-33.0)	-	32.0 (31.0-33.0)
Female	33.0 (31.0-33.0)	32.0 (30.0-33.0)	32.0	30.0 (24.0-32.0)	32.0 (30.0-33.0)	31.0 (31.0-32.0)	31.0 (31.0-31.0)	32.0 (31.0-32.0)
Apgar score, median (IQR)[j,k]								
1 min								
Male	9	8 (8-9)	8 (7-8)	6	8 (7-9)	8 (7-8)	-	9 (6-9)
Female	9 (8-9)	9 (8-9)	8	8 (8-8)	8 (8-9)	8 (8-9)	8 (7 -9)	8 (8-8)
5 min								
Male	9	9 (9-9)	9 (9-9)	8	9 (9-10)	9 (8-9)	-	9 (9-10)
Female	9 (9-10)	9 (9-10)	9	9 (9-9)	9 (9-10)	9 (9-10)	10 (9-10)	9 (9-9)

N, number of individuals; y, years; w, weeks; SD, standard deviation; IQR, interquartile range; No., number of events; Mixed infection – *P. vivax*- and *P. falciparum*-infection occurring at the same time or at different times during pregnancy.

[a] Maternal age in the Retrospective Cohort Study was recorded in 238 non-infected pregnant women.
[b] Gravidity in the Retrospective Cohort Study was recorded in 235 non-infected and 8 *P. falciparum*-infected pregnant women.
[c] Maternal weight gain was recorded in 4 non-infected, 12 *P. vivax,* 2 mixed-infected and 3 *P. falciparum* pregnant women. It was determined by subtracting the initial pregnancy weight from the final weight.
[d] Hematocrit was recorded in 4 non-infected, 10 *P. vivax*, 3 mixed-infected and 2 *P. falciparum* pregnant women.
[e] Hemoglobin was recorded in 4 non-infected, 10 *P. vivax*, 3 mixed-infected and 2 *P. falciparum* pregnant women.
[f] Placental weight was recorded in 3 non-infected, 12 *P. vivax,* and 3 mixed-infected pregnant women.
[g] The number of antenatal care visits in the Prospective Cohort Study was recorded in 13 *P. vivax*-infected pregnant women, and in the Retrospective Cohort Study in 218 non-infected, 20 *P. vivax* and 7 *P. falciparum*-infected pregnant women.
[h] Length and Rohrer index in the Retrospective Cohort Study was recorded in 241 newborns from non-infected pregnant women
[i] The Rohrer index is the newborns' weight in grams divided by the cube of the length in centimeters, and newborns are considered proportional when values are between 2.32-2.85.
[j] Apgar score: 7 – 10, normal; 4 – 6, some breathing assistance might be required; and, < 4, more assistance must be provided.
[k] Apgar score at 1 and 5 minutes in the Prospective Cohort Study were recorded in 13 *P. vivax* and 3 mixed-infected pregnant women, and in the Retrospective Cohort Study in 243 newborns from non-infected pregnant women.

© 2019 Dombrowski JG et al. *JAMA Network Open*.

eTable 3. Baseline Characteristics of Newborns at Delivery of Non-Infected and *P. Falciparum*-Infected Pregnant Women of the Prospective Cohort Study

Newborns' Characteristics	Median (IQR)				
	**Non-Infected (N:M=59, F=79)**	**NI-SH (N:M=13, F=7)**	***Pf*-NHC (N:M=49, F=45)**	***Pf*-SH (N:M=15, F=15)**	***Pf*-MC (N:M=3, F=5)**
Weight, g					
Male					
Mean (SD)	3301.7 (353.3)	2985.8 (227.5)	3351.1 (328.0)	3067.3 (414.7)	3275.0 (888.1)
Median (IQR)	3305.0 (3045.0-3530.0)	2900.0 (2800.0-3125.0)	3320.0 (3135.0-3530.0)	3030.0 (2790.0-3160.0)	2790.0 (2735.0-4300.0)
Female					
Mean (SD)	3394.9 (421.6)	3017.1 (246.9)	3217.9 (346.8)	2918.3 (247.9)	2897.0 (142.3)
Median (IQR)	3400.0 (3055.0-3690.0)	3000.0 (2890.0-3240.0)	3170.0 (2960.0-3390.0)	2910.0 (2700.0-3100.0)	2905.0 (2870.0-2910.0)
Length, cm[a]					
Male					
Mean (SD)	49.4 (1.4)	48.8 (1.2)	49.6 (1.9)	49.3 (1.6)	50.3 (3.2)
Median (IQR)	49.0 (48.0-50.0)	48.0 (48.0-49.0)	50.0 (48.0-51.0)	49.0 (48.0-50.0)	49.0 (48.0-54.0)
Female					
Mean (SD)	49.6 (1.4)	48.4 (1.7)	49.2 (1.5)	48.3 (2.1)	48.6 (2.1)
Median (IQR)	50.0 (49.0-50.0)	48.0 (47.0-50.0)	49.0 (48.0-50.0)	48.0 (47.0-50.0)	48.0 (47.0-49.0)
Rohrer index[a, b]					
Male					
Mean (SD)	2.75 (0.28)	2.58 (0.19)	2.76 (0.29)	2.55 (0.16)	2.52 (0.2)
Median (IQR)	2.79 (2.56-2.96)	2.55 (2.41-2.67)	2.71 (2.57-2.95)	2.52 (2.42-2.69)	2.52 (2.32-2.73)
Female					
Mean (SD)	2.78 (0.27)	2.67 (0.34)	2.67 (0.24)	2.60 (0.31)	2.55 (0.4)
Median (IQR)	2.76 (2.58-2.93)	2.71 (2.39-2.97)	2.67 (2.52-2.90)	2.58 (2.34-2.80)	2.63 (2.29-2.80)
Head circumference, cm					
Male					
Mean (SD)	34.8 (1.1)	32.6 (0.7)	34.6 (1.0)	32.4 (0.7)	31.7 (0.6)
Median (IQR)	35.0 (34.0-36.0)	33.0 (32.0-33.0)	35.0 (34.0-35.0)	32.0 (32.0-33.0)	32.0 (31.0-32.0)
Female					
Mean (SD)	34.5 (1.1)	31.9 (0.7)	34.3 (0.9)	31.3 (1.0)	30.2 (0.8)
Median (IQR)	34.0 (34.0-35.0)	32.0 (31.0-32.5)	34.0 (34.0-35.0)	32.0 (31.0-32.0)	30.0 (30.0-31.0)
Apgar score[c, d]					
1 min					
Male					
Mean (SD)	8.1 (1.4)	8.8 (0.5)	8.2 (1.0)	8.5 (0.9)	8.5 (0.7)
Median (IQR)	8.0 (8.0-9.0)	9.0 (8.5-9 0)	8.0 (8.0-9.0)	9.0 (8.0-9.0)	8.5 (8.0-9.0)

© 2019 Dombrowski JG et al. *JAMA Network Open*.

eTable 3. Baseline characteristics of newborns at delivery of non-Infected and *P. falciparum*-infected pregnant women of the Prospective Cohort Study (continued).

Newborns' Characteristics	Median (IQR)				
	**Non-Infected (N:M=59, F=79)**	**NI-SH (N:M=13, F=7)**	***Pf*-NHC (N:M=49, F=45)**	***Pf*-SH (N:M=15, F=15)**	***Pf*-MC (N:M=3, F=5)**
Female					
Mean (SD)	8.5 (0.8)	7.9 (1.3)	8.3 (0.9)	8.8 (0.5)	8.8 (0.4)
Median (IQR)	9.0 (8.0-9.0)	8.0 (6.0-9.0)	8.0 (8.0-9.0)	9.0 (8.5-9.0)	9.0 (9.0-9.0)
5 min					
Male					
Mean (SD)	9.2 (0.9)	9.7 (0.5)	9.4 (0.6)	9.6 (0.6)	9.5 (0.7)
Median (IQR)	9.0 (9.0-10.0)	10.0 (9.0-10.0)	9.0 (9.0-10.0)	10.0 (9.0-10.0)	9.5 (9.0-10.0)
Female					
Mean (SD)	9.5 (0.5)	9.4 (0.5)	9.5 (0.6)	9.8 (0.5)	9.8 (0.4)
Median (IQR)	9.0 (9.0-10.0)	9.0 (9.0-10.0)	9.0 (9.0-10.0)	10.0 (9.5-10.0)	10.0 (10.0-10.0)

IQR, interquartile range; N, number of newborns; M, male newborns; F, female newborns, NI-SH, Non-Infected small head; *Pf*-NHC, *Plasmodium falciparum*-normal head circumference; *Pf*-SH, *Plasmodium falciparum*-small head; *Pf*-MC, *Plasmodium falciparum*-microcephaly.

[a] Length and Rohrer index were recorded in 58 males from the Non-infected group.

[b] The Rohrer index is the newborns' weight in grams divided by the cube of the length in centimeters, and newborns are considered proportional when values are between 2.32 - 2.85.

[c] Apgar score was recorded in 57 males and 77 females from the Non-infected group; in 12 males from the NI-SH group; in 45 males and 39 females from the Pf-NHC group; in 14 males and 12 females from the Pf-SH group; and, in 2 males from the Pf-MC group.

[d] Apgar score: 7 – 10, normal; 4 – 6, some breathing assistance might be required; and, < 4, more assistance must be provided.

© 2019 Dombrowski JG et al. *JAMA Network Open*.

eTable 4. Summary of the Serological Screening of TORCH, HIV, Syphilis, Dengue, Chikungunya and Zika Infections

Infectious Agent	Kit validity range OD[a] (batch)	Obtained Standard OD	OD interpretation	Manufacturer
**TORCH / IgM**				
Toxoplasma gondii	0.38-1.29 (SHF.AQ)	1.200	<0.61 Negative<br>0.61-0.71 Borderline<br>>0.71 Positive	SERION®Immunologics, Germany
Rubella	0.43-1.46 (SAG.BS)	0.96	<0.28 Negative<br>0.28-0.38 Borderline<br>>0.38 Positive	SERION®Immunologics, Germany
Cytomegalovirus	0.46-1.55 (SEF.BZ)	1.310	<0.91 Negative<br>0.91-1.16 Borderline<br>>1.16 Positive	SERION®Immunologics, Germany
Herpes simplex virus (type 2)	0.44-1.50 (SGF.BQ)	1.500	<1.60 Negative<br>1.60–2.16 Borderline<br>>2.16 Positive	SERION®Immunologics, Germany
**TORCH / IgG**				
Toxoplasma gondii	0.45-1.51 (SHF.AK)	1.043	<0.14 Negative<br>0.14-0.25 Borderline<br>>0.25 Positive	SERION®Immunologics, Germany
Rubella	0.45-1.53 (SDF.FA)	0.91	<0.35 Negative<br>0.35-0.59 Borderline<br>>0.59 Positive	SERION®Immunologics, Germany
Cytomegalovirus	0.45-1.53 (SBF.HA)	1.349	<0.52 Negative<br>0.52-0.73 Borderline<br>>0.73 Positive	SERION®Immunologics, Germany
Herpes simplex virus (type 2)	0.47-1.60 (SDFAS)	1.389	<0.21 Negative<br>0.21-0.30 Borderline<br>>0.30 Positive	SERION®Immunologics, Germany
**OTHER RELEVANT INFECTIONS[b]**				
Syphilis	Negative <0.10<br>Positive ≥1.00<br>(1003000411)	Negative =0.01<br>Positive =2.48	<0.8 Negative<br>0.8-1.2 Borderline<br>>1.2 Positive	Symbiosys, São Paulo, Brazil
HIV[c]	Negative <0.20<br>Positive ≥0.80<br>(1000000630)	Negative =0.01<br>Positive =2.51	<0.9 Negative<br>0.9-1 Borderline<br>>1 Positive	Symbiosys, São Paulo, Brazil
**ARBOVIRUSES**				
Dengue virus				
IgG			<0.063 Negative<br>0.083-0.063 Borderline<br>>0.083 Positive	University of São Paulo, Brazil
IgM			<0.2 Negative<br>≥0.2 Positive	University of São Paulo, Brazil
Chikungunya virus				
IgG			<0.2 Negative<br>≥0.2 Positive	Institute Pasteur Dakar, Senegal
IgM			<0.2 Negative<br>≥0.2 Positive	Institute Pasteur Dakar, Senegal
Zika virus				
IgG			**DENV (-)**<br><0.219 Negative<br>0.331-0.219 Borderline<br>>0.331 Positive<br><br>**DENV (+)**<br><0.365 Negative<br>0.533-0.365 Borderline	Institute Pasteur Dakar, Senegal

© 2019 Dombrowski JG et al. *JAMA Network Open*.

			>0.533 Positive	
IgM			<0.2 Negative<br>≥0.2 Positive	Institute Pasteur Dakar, Senegal

OD, optical density.

[a] The kit validity range is according to the batch.

[b] Total antibodies. The validity range was verified through a negative and positive standard OD obtained in each test.

[c] Isotypes 1 and 2.

© 2019 Dombrowski JG et al. *JAMA Network Open*.

eTable 5. Results of the Screening of Other Infectious Agents in Mothers of Newborns With Small Head in the Prospective Cohort

Infectious Agent	IgG+ (N=87)			IgG/IgM+ (N=87)			IgM+ (N=87)			Avidity[a] (N=31)			Excluded[b]
	**NI (N=31)**	***Pv* (N=31)**	***Pf* (N=25)**	**NI (N=31)**	***Pv* (N=31)**	***Pf* (N=25)**	**NI (N=31)**	***Pv* (N=31)**	***Pf* (N=25)**	**NI (N=31)**	***Pv* (N=31)**	***Pf* (N=25)**	
Toxoplasma gondii[c]	15	16	14	1	3	4	3	0	2	-	-	-	5
Confirmation[d]													1
T1	-	-	8	-	-	2	-	-	2	-	-	1	
T2[e]	-	-	8	-	-	3	-	-	0	-	-	-	
Rubella[c]	21	21	10	6	6	5	1	1	2	-	-	-	4
Confirmation[d]													0
T1	-	-	8	-	-	4	-	-	0	-	-	0	
T2[e]	-	-	8	-	-	3	-	-		-	-	-	
Cytomegalovirus[c]	31	30	25	0	0	0	0	0	0	-	-	-	0
Confirmation[d]													0
T1	-	-	16	-	-	0	-	-	0	-	-	-	
T2[e]	-	-	15	-	-	-	-	-	-	-	-	-	
Herpes simplex virus (type 2) [c]	9	6	10	0	0	0	1	0	0	-	-	-	1
Confirmation[d]													2
T1	-	-	5	-	-	0	-	-	2	-	-	-	
T2[e]	-	-	6	-	-	-	-	-	0	-	-	-	
Syphilis[c]	0	0	0	0	0	0	0	1	1	-	-	-	2
HIV[c]	0	0	0	0	0	0	0	0	0	-	-	-	0
Dengue virus[c, f]	6	6	14	0	0	0	0	0	0	-	-	-	0
Chikungunya virus[c, f]	0	0	0	0	0	0	0	0	0	-	-	-	0
Zika virus[c, f]	0	0	1[g]	0	0	0	0	0	0	-	-	-	0

NI, non-infected; *Pv*, *Plasmodium vivax*-infected women during pregnancy; *Pf*, *P. falciparum*-infected women during pregnancy, irrespective of newborns' head circumference; N, number of individuals.

[a] The interpretation of the avidity test was made accordingly to manufacturers' recommendations: high avidity (>50%) indicates a past infection that occurred more than 4 months; low avidity (<45%) indicates recent infection, less than three months.

[b] Samples were excluded whenever that sample was IgM positive and presented low avidity.

[c] The initial screening was performed in samples acquired between 16th and 30th gestation week. In the case of pregnant women that we do not have samples from this window, were used samples collected close to that period.

[d] Confirmation was executed in all pregnant women that were only IgM positive for at least one infectious agent. The confirmation was performed in two different time-points: sample obtained during the 1st trimester (T1) and sample obtained during the 3rd trimester (T2), followed by an avidity test.

[e] One sample was only tested in one time-point.

[f] Tested only in 8 NI, 10 *Pv,* and 19 *Pf* for IgG; and 8 NI, 10 *Pv,* and 19 *Pf* for IgM.

[g] The sample that was IgG positive for Zika virus was considered a possible cross-reaction with another flavivirus, as the absorbance levels were at borderline. Until 2016 there were no reported cases of Zika virus infection in Acre state.

© 2019 Dombrowski JG et al. *JAMA Network Open*.

eTable 6. Placental Histological Parameters and Angiogenic Factors of Non-Infected And *P. Falciparum*-Infected Pregnant Women of the Prospective Cohort Study

Characteristics	Median (IQR)							
	**Non-Infected (N=128)**	**Non-Infected-SH (N=20)**	***P* Value[a]**	***Pf*-NHC (N=80)**	***P* Value[b]**	***Pf*-SH (N=24)**	***P* Value[c]**	***Pf*-MC (N=7)**	***P* Value[d]**
**Placental histological parameters**									
Leukocytes infiltrate[e]	16.0 (9.0-21.0)	14.0 (10.5-24.0)	> .99	20.0 (13.0-31.0)	.008	18.0 (14.0-35.0)	.39	18.0 (17.0-52.0)	.08
Monocytes infiltrate[f]	4.0 (2.0-7.0)	3.0 (2.0-4.5)	> .99	7.0 (5.0-13.0)[g]	< .001	9.5 (5.5-15.0)[h]	< .001	9.0 (6.0-11.0)	.02
Fibrin deposition score	1.9 (1.9-2.4)	1.9 (1.9-2.8)	> .99	2.4 (1.9-2.8)	< .001	2.0 (1.9-2.8)	> .99	2.1 (1.9-2.8)	.56
Fibrinoid necrosis[i]	6.0 (3.8-10.2)	7.2 (4.5-10.3)	> .99	7.1 (4.3-10.0)	> .99	6.8 (4.0-9.8)	> .99	8.1 (2.9-9.4)	.94
Proliferation index[j]	3.6 (2.5-4.9)	3.6 (2.5-4.4)	> .99	3.8 (2.8-4.8)	> .99	3.7 (2.9-4.2)	> .99	3.0 (2.6-3.5)	.21
Villous vascularity[k]	4.0 (3.6-4.5)	3.9 (3.4-4.4)	> .99	4.0 (3.4-4.5)	> .99	4.1 (3.6-4.6)	> .99	3.8 (3.6-3.9)	.35
Syncytial nuclear aggregates	13.0 (10.0-17.0)	14.5 (9.5-16.5)	> .99	13.5 (10.0-19.0)	> .99	17.5 (12.0-24.5)	.01	18.0 (12.0-30.0)	.02
**Angiogenic factors, ng/mL[l]**									
ANG-1[m]	14.9 (10.2-21.1)	20.8 (14.2-28.8)	.16	17.7 (10.6-25.9)	.28	18.1 (11.2-22.3)	> .99	19.4 (10.4-51.7)	.47
ANG-2	7.0 (3.5-12.8)	4.6 (3.0-11.5)	> .99	4.2 (1.8-9.1)	.04	5.5 (2.2-10.0)	.52	5.1 (0-8.9)	.12
ANG-2/ANG-1 ratio	0.4 (0.2-1.0)	0.3 (0.1-.7)	.65	0.3 (0.1-0.7)	.06	0.2 (0.1-0.8)	.55	0.2 (0-0.5)	.08
Tie-2	13.2 (8.7-18.1)	16.6 (14.8-21.2)	.47	16.2 (12.2-21.9)	.01	15.1 (11.7-21.7)	.41	18.5 (11.8-22.1)	.15
Tie-2/ANG-1 ratio	0.9 (0.6-1.3)	0.7 (0.6-1.1)	> .99	0.9 (0.6-1.5)	> .99	0.9 (0.7-1.5)	> .99	0.9 (0.4-2.7)	.94
VEGF[m]	3.2 (1.5-4.6)	4.3 (2.5-5.4)	.17	3.7 (2.4-4.5)	.57	2.9 (1.6-4.7)	> .99	4.8 (4.5-5.7)	.009
VEGFR1	89.6 (38.9-123.3)	60.4 (11.5-121.3)	> .99	81.1 (16.4-133.1)	> .99	74.7 (13.0-121.8)	> .99	54.2 (17.2-60.6)	.07
VEGFR-2	14.2 (12.7-15.8)	14.7 (13.5-15.6)	> .99	14.4 (13.3-15.7)	> .99	14.3 (12.3-16.2)	> .99	13.6 (12.4-16.1)	.61
**Leptin, ng/mL[n]**	33.1 (17.2-47.4)	22.5 (10.7-37.5)	> .99	21.2 (13.8-42.3)	.28	19.5 (4.5-37.2)	.06	16.7 (9.0-26.7)	.03

IQR, interquartile range; N, number of individuals; Non-Infected-SH, non-infected-small head; *Pf*-NHC, *Plasmodium falciparum*-normal head circumference; *Pf*-SH, *Plasmodium falciparum*-small head; *Pf*-MC, *Plasmodium falciparum*-microcephaly. Differences between Non-Infected, Non-Infected-SH, *Pf*-NHC and *Pf*-SH groups were evaluated using the Kruskal-Wallis rank test with Dunn's post-test.

[a] Differences between Non-Infected and Non-Infected-SH groups.
[b] Differences between Non-Infected and *Pf*-NHC groups.
[c] Differences between Non-Infected and *Pf*-SH groups.
[d] Differences between *Pf*-MC and other groups were evaluated using the Mann-Whitney rank sum test. The values presented are the differences between Non-Infected and *Pf*-MC groups.
[e] Leukocyte infiltrate (CD45+) was recorded in placentas from 126 non-infected, 54 *Pf*-NHC, 17 *Pf*-SH, and 5 *Pf*-MC pregnant women.
[f] Monocytes infiltrate (CD68+) was recorded in placentas from 127 non-infected pregnant women.
[g] Statistical difference for the comparison of Non-Infected-SH versus *Pf*-NHC, P < .001.
[h] Statistical difference for the comparison of Non-Infected-SH versus *Pf*-SH, P < .001.
[i] Fibrinoid necrosis was recorded in placentas from 78 *Pf*-NHC pregnant women.
[j] Proliferation index was recorded in placentas from 126 non-infected and 77 *Pf*-NHC pregnant women.
[k] Villous vascularity was recorded in placentas from 124 non-infected, 71 *Pf*-NHC, and 23 *Pf*-SH pregnant women.
[l] Angiogenic factors were recorded in placental plasma from 126 non-infected, 18 non-infected-SH, and 79 *Pf*-NHC pregnant women. VEGF denotes vascular endothelial growth factor A, VEGFR1 and VEGFR-2 vascular endothelial growth factor A receptor 1 and 2, Ang-1 and 2 angiopoietin-1 and 2.

© 2019 Dombrowski JG et al. *JAMA Network Open*.

[m] ANG-1 and VEGF were recorded in placental plasma from 19 non-infected-SH pregnant women.
[n] Leptin was recorded in placental plasma from 126 non-infected, 18 non-infected-SH, 77 *Pf*-NHC, and 23 *Pf*-SH pregnant women.

© 2019 Dombrowski JG et al. *JAMA Network Open*.

eTable 7. Baseline characteristics of Mothers and Newborns of the Retrospective Cohort Study

Characteristics	Non-Infected (N=3650)	*P. vivax* (N=129)	Mixed (N=21)	*P. falciparum* (N=82)
**Mothers**				
Maternal age, mean (SD), y [a]	24.1 (6.4)	22.7 (6.1)	23.4 (6.4)	24.1 (6.6)
Gravidity, No. (%)[b]				
Primigravida	1286 (36.4)	46 (36.2)	6 (28.6)	22 (28.2)
Multigravida	2247 (63.6)	81 (63.8)	15 (71.4)	56 (71.8)
Gestational age at delivery, w				
Mean (SD)	39.3 (1.1)	39.0 (1.2)	39.2 (1.4)	39.0 (1.1)
Median (IQR)	39.0 (38.0-40.0)	39.0 (38.0-40.0)	39.0 (38.0-40.0)	39.0 (38.0-40.0)
C-section, No. (%)	1283 (35.2)	43 (33.3)	7 (33.3)	22 (26.8)
Antenatal care visits, mean (SD)[c]	5.9 (2.5)	5.8 (2.4)	5.6 (2.4)	5.5 (2.8)
**Newborns**				
Male newborns, No. (%)	1921 (52.6)	67 (51.9)	10 (47.6)	47 (57.3)
Weight, g				
Male				
Mean (SD)	3372.5 (423.1)	3362.2 (418.1)	3371.5 (382.3)	3234.5 (391.6)
Median (IQR)	3325.0 (3065.0-3650.0)	3360.0 (3005.0-3600.0)	3242.5 (3165.0-3750.0)	3180.0 (2960.0-3470.0)
Female				
Mean (SD)	3239.0 (381.0)	3161.8 (328.8)	3013.2 (341.4)	3176.1 (447.5)
Median (IQR)	3200.0 (2965.0-3480.0)	3150.0 (2910.0-3410.0)	3035.0 (2700.0-3340.0)	3100.0 (2860.0-3380.0)
Length, cm[d]				
Male				
Mean (SD)	49.5 (1.9)	49.2 (1.8)	49.4 (1.8)	48.9 (1.9)
Median (IQR)	49.0 (48.0-51.0)	49.0 (48.0-50.0)	49.0 (48.0-51.0)	49.0 (48.0-50.0)
Female				
Mean (SD)	48.9 (1.8)	48.5 (1.9)	49.1 (2.0)	48.5 (1.7)
Median (IQR)	49.0 (48.0-50.0)	48.0 (48.0-49.0)	49.0 (48.0-50.0)	48.0 (47.0-49.0)
Rohrer index[d, e]				
Male				
Mean (SD)	2.8 (0.3)	2.8 (0.3)	2.8 (0.5)	2.8 (0.3)
Median (IQR)	2.8 (2.6-3.0)	2.8 (2.6-3.0)	2.8 (2.5-3.0)	2.8 (2.6-3.0)
Female				
Mean (SD)	2.8 (0.3)	2.8 (0.3)	2.6 (0.3)	2.8 (0.4)
Median (IQR)	2.8 (2.6-3.0)	2.8 (2.6-2.9)	2.4 (2.3-2.7)	2.7 (2.5-3.0)
Head circumference, cm				
Male				
Mean (SD)	34.4 (1.6)	34.2 (1.3)	34.4 (1.8)	33.7 (1.4)
Median (IQR)	34.0 (33.0-35.0)	34.0 (33.0-35.0)	34.5 (33.0-36.0)	34.0 (33.0-35.0)
Female				
Mean (SD)	33.9 (1.5)	33.6 (1.5)	33.5 (1.6)	33.7 (1.6)
Median (IQR)	34.0 (33.0-35.0)	34.0 (33.0-35.0)	34.0 (32.0-35.0)	33.0 (32.0-35.0)
Apgar score[f, g]				
1 min				
Male				
Mean (SD)	8.4 (1.0)	8.6 (0.6)	8.0 (1.2)	8.4 (1.2)
Median (IQR)	9 (8-9)	9 (8-9)	8 (8-9)	9 (8-9)
Female				
Mean (SD)	8.4 (0.9)	8.3 (1.2)	8.5 (0.7)	8.3 (0.9)
Median (IQR)	9 (8-9)	9 (8-9)	9 (8 -9)	8 (8-9)
5 min				
Male				
Mean (SD)	9.5 (0.6)	9.6 (0.5)	9.3 (0.7)	9.6 (0.6)
Median (IQR)	10 (9-10)	10 (9-10)	9 (9-10)	10 (9-10)

© 2019 Dombrowski JG et al. *JAMA Network Open*.

eTable 7. Baseline characteristics of mothers and newborns of the Retrospective Cohort Study (continued).

Characteristics	Non-Infected (N=3650)	*P. vivax* (N=129)	Mixed (N=21)	*P. falciparum* (N=82)
Female				
Mean (SD)	9.5 (0.6)	9.4 (0.8)	9.5 (0.5)	9.5 (0.5)
Median (IQR)	10 (9-10)	10 (9-10)	10 (9-10)	10 (9-10)

N, number of individuals; SD, standard deviation; IQR, interquartile range; No., number of events; Mixed infection – *P. vivax*- and *P. falciparum*-infection occurring at the same time and at different times during pregnancy.

[a] Maternal age was recorded in 3372 non-infected and 126 *P. vivax*-infected pregnant women.

[b] Gravidity was recorded in 3533 non-infected, 127 *P. vivax,* and 78 *P. falciparum*-infected pregnant women.

[c] The number of antenatal care visits was recorded in 3413 non-infected, 125 *P. vivax*, 20 mixed-infected and 79 *P. falciparum* pregnant women.

[d] Length and Rohrer index was recorded in 3635 newborns from non-infected pregnant women.

[e] The Rohrer index is the newborns' weight in grams divided by the cube of the length in centimeters, and newborns are considered proportional when values are between 2.32-2.85.

[f] Apgar score: 7 – 10, normal; 4 – 6, some breathing assistance might be required; and, <4, more assistance must be provided.

[g] Apgar score at 1 and 5 minutes was recorded in 3628 newborns from non-infected and 81 *P. falciparum*-infected pregnant women.

© 2019 Dombrowski JG et al. *JAMA Network Open*.

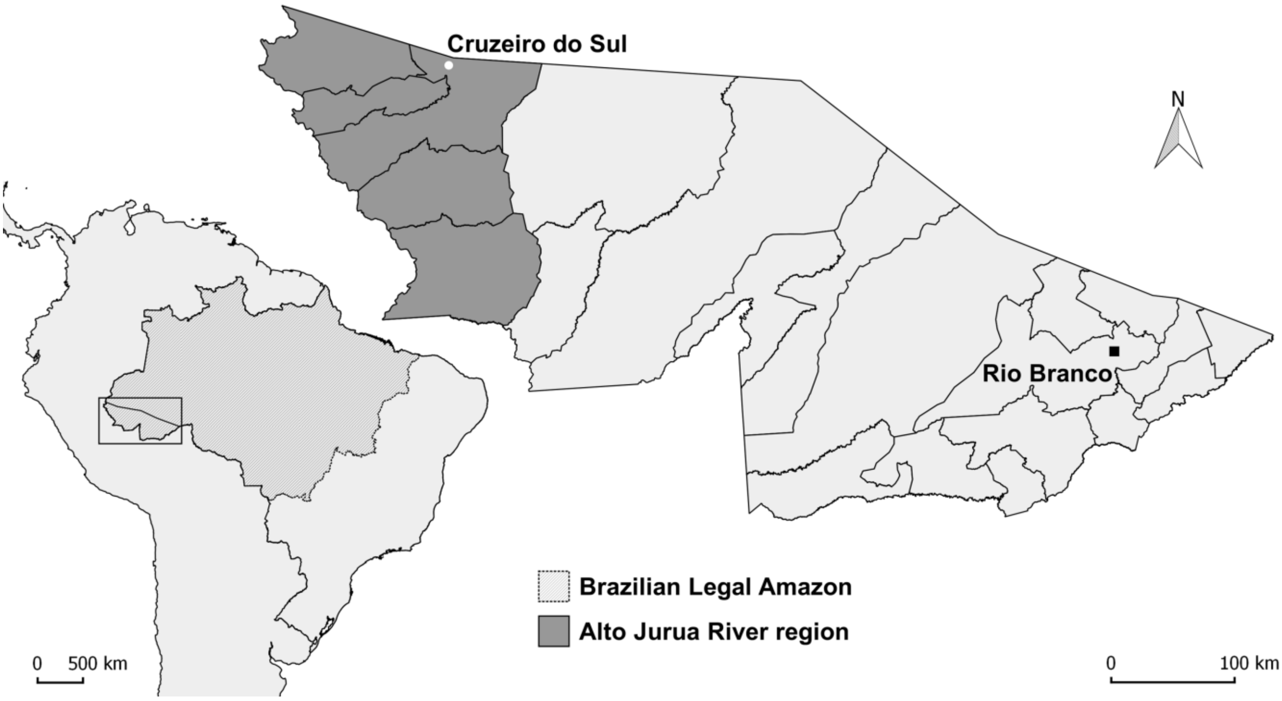

eFigure 1. Map Showing the Location of the Field Site, Alto do Juruá Region, Northwest of the Acre State, Brazilian Amazon

The map also indicates Cruzeiro do Sul where the field laboratory is situated, and Rio Branco, the capital of the state of Acre.

© 2019 Dombrowski JG et al. *JAMA Network Open*.

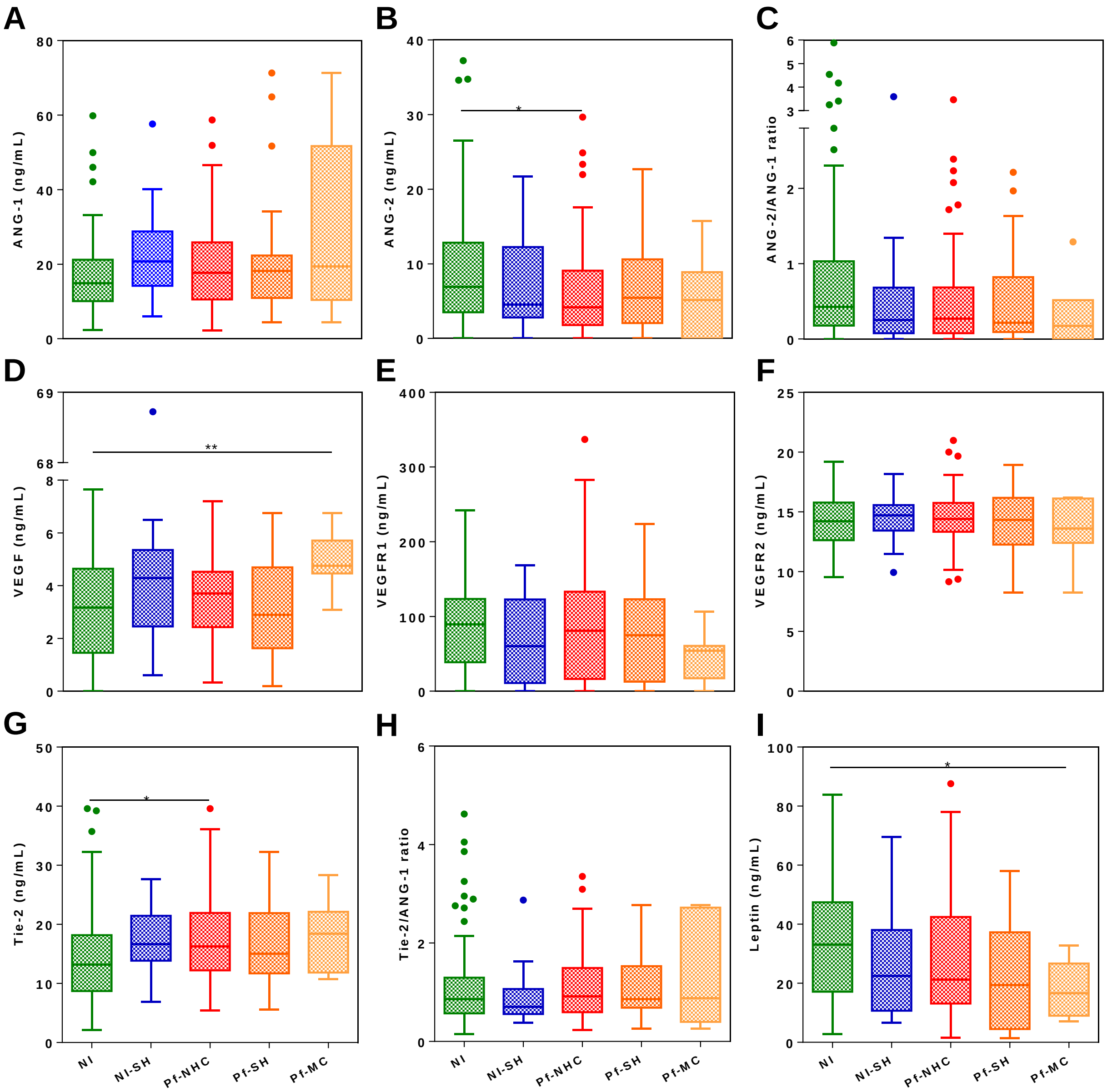

eFigure 2. Placental Plasma Levels of Angiogenic Factors and Leptin From Non- and *P. Falciparum*-Infected Mothers According to Newborns Head Circumference. **A** Angiopoietin-1 (ANG-1). **B** Angiopoietin-2 (ANG-2). **C** Ratio ANG-2/ANG-1. **D** Vascular endothelial growth factor (VEGF). **E** VEGF receptor-1 (VEGFR-1). **F** VEGF receptor-2 (VEGFR-2). **G** TEK receptor tyrosine kinase (Tie-2). **H** Ratio Tie-2/ANG-2. **I** Leptin. All factors were measured by ELISA. NI – non-infected (N=126); NI-SH – non-infected small head (N=18-19); Pf-NHC – *P. falciparum*-infected normal head circumference (N=77-79); Pf-SH - *P. falciparum*-infected small head (N=23-24); and, Pf-MC - *P. falci*parum-infected microcephaly (N=7). Data are represented as Tukey boxplots, the bottom and the top of the box are the first and third quartiles, the line inside the box is the median, and the whiskers represent the lowest and the highest data within 1.5xIQR of the first and upper quartiles. The differences between each group were determined by Kruskal-Wallis test with Dunn's post hoc correction, * P < .05, ** P < .01.

© 2019 Dombrowski JG et al. *JAMA Network Open*.

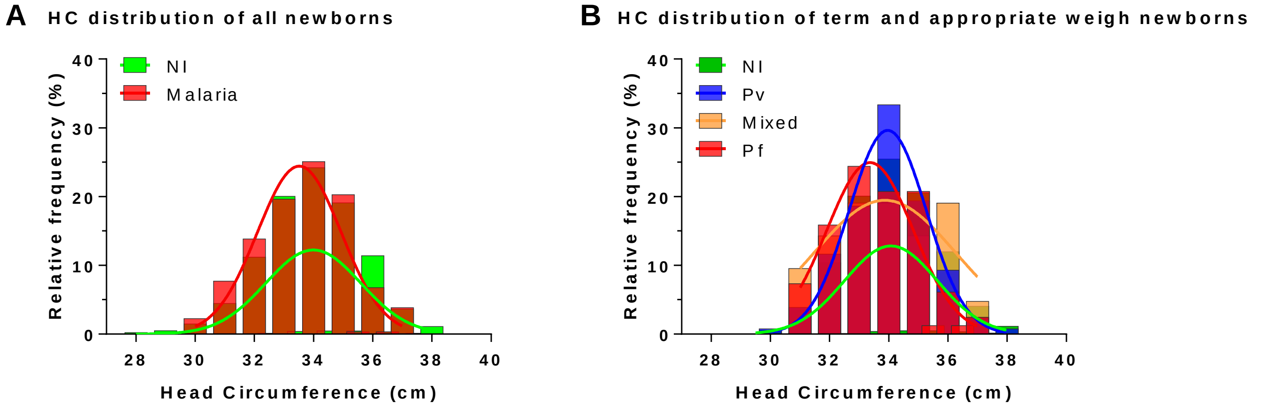

C Forest plot of reduction of head circumference ocurrence likelihood

Newborns	n/N	Prevalence (%)	Odds Ratio (95% CI)	*P*-Value
**Small Head (HC < -1 SD)**	934/3882	24.1		
P. vivax	32/129	24.8	1.03 (0.68 – 1.56)	0.88
Mixed	7/21	33.3	1.61 (0.64 – 4.05)	0.31
P. falciparum	30/82	36.6	1.91 (1.21 – 3.04)	0.006
**Microcephaly (HC < -2 SD)**	161/3882	4.2		
P. vivax	6/129	4.7	1.02 (0.43 - 2.40)	0.96
Mixed	2/21	9.5	2.41 (0.52 – 11.24)	0.26
P. falciparum	6/82	7.3	2.14 (0.89 – 5.19)	0.09

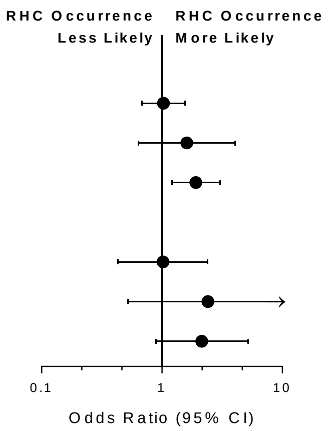

eFigure 3. Retrospective Cohort Study Supports That Malaria Infection During Pregnancy Impacts Newborns Head Circumference.

A, B Newborns head circumference frequency distribution in the PCS according to maternal infection status: malaria- and non-infected (NI) mothers (*P* = .008) (**A**), and NI, *Pv*, Mixed and *Pf*-infected mothers after excluding LBW and preterm newborns (NI vs *Pf P* = .04) (**B**). A, NI – N = 3889 and HC mean = 33.92; Malaria – N = 311 and HC mean = 33.67. B, NI – N = 3650 and HC mean = 34.12; *Pv* – N = 129 and HC mean = 33.92; Mixed – N = 21 and HC mean = 33.91; *Pf* – N = 82 and HC mean = 33.67.The differences between each group were determined by examining with Mann-Whitney rank sum tests (A) and Kruskal-Wallis test with Dunn's post hoc correction (B). **C** Forest plot of the Odds Ratio of small head or microcephaly in newborns from women infected during pregnancy compared to newborns from non-infected women, according to *Plasmodium* species. Mixed infection denotes *P. vivax*- and *P. falciparum*-infection occurring at the same time and at different times during pregnancy. n/N - number of events by total number of individuals in each group; CI - confidence interval; HC - head circumference; RHC - reduction of the head circumference; SD - standard deviation; p values were estimated through multivariate logistic regression methods.

© 2019 Dombrowski JG et al. *JAMA Network Open*.

eReferences

1 Lucchi NW, Narayanan J, Karell MA, *et al.* Molecular Diagnosis of Malaria by Photo-Induced Electron Transfer Fluorogenic Primers: PET-PCR. *PLoS One* 2013; **8**: e56677.

2 Gravidez RIANA, Crian NA, Meses MDES. Esquemas recomendados para malária não complicada. ; : 4–8.

3 Souza RM, Ataíde R, Dombrowski JG, *et al.* Placental Histopathological Changes Associated with Plasmodium vivax Infection during Pregnancy. *PLoS Negl Trop Dis* 2013; **7**. DOI:10.1371/journal.pntd.0002071.

4 Romagosa C, Menendez C, Ismail MR, *et al.* Polarisation microscopy increases the sensitivity of hemozoin and Plasmodium detection in the histological assessment of placental malaria. *Acta Trop* 2004; **90**: 277–84.

5 Ataíde R, Murillo O, Dombrowski JG, *et al.* Malaria in Pregnancy Interacts with and Alters the Angiogenic Profiles of the Placenta. *PLoS Negl Trop Dis* 2015; **9**: e0003824.

6 Hsu SM, Raine L, Fanger H. A comparative study of the peroxidase-antiperoxidase method and an avidin-biotin complex method for studying polypeptide hormones with radioimmunoassay antibodies. *Am J Clin Pathol* 1981; **75**: 734–8.

7 Tuominen VJ, Ruotoistenmaki S, Viitanen A, Jumppanen M, Isola J. ImmunoRatio: a publicly available web application for quantitative image analysis of estrogen receptor (ER), progesterone receptor (PR), and Ki-67. *Breast Cancer Res* 2010; **12**: R56.

8 Sow A, Loucoubar C, Diallo D, *et al.* Concurrent malaria and arbovirus infections in Kedougou, southeastern Senegal. *Malar J* 2016; **15**: 47.

9 Umbers AJ, Aitken EH, Rogerson SJ. Malaria in pregnancy: small babies, big problem. *Trends Parasitol* 2011; **27**: 168–75.

10 WHO Expert Committee on Physical Status. Physical status: the use and interpretation of anthropometry. Geneva: World Health Organization, 1995.

11 American Academy of Pediatrics Committee on Fetus and Newborn, American College of Obstetricians and Gynecologists Committee on Obstetric Practice. The Apgar Score. *Pediatrics* 2015; **136**: 819.

12 Avery JW, Smith GM, Owino SO, *et al.* Maternal malaria induces a procoagulant and antifibrinolytic state that is embryotoxic but responsive to anticoagulant therapy. *PLoS One* 2012; **7**: e31090.

13 Maley SW, Buxton D, Macaldowie CN, *et al.* Characterization of the immune response in the placenta of cattle experimentally infected with Neospora caninum in early gestation. *J Comp Pathol* 2006; **135**: 130–41.

© 2019 Dombrowski JG et al. *JAMA Network Open*.
